# Safety and immunogenicity testing of a melioidosis subunit vaccine candidate in cynomolgus macaques

**DOI:** 10.1038/s41541-026-01526-5

**Published:** 2026-07-14

**Authors:** Sineenart Sengyee, Caitlyn E. Orne, Sarah B. Weiby, Lindsey K. Schmidt, Federico Urbano-Munoz, Jiri Vlach, Christian Heiss, Parastoo Azadi, Narisara Chantratita, Mary N. Burtnick, Paul J. Brett

**Affiliations:** 1 Department of Microbiology and Immunology, University of Nevada, Reno School of Medicine, Reno, NV USA; 2 Complex Carbohydrate Research Center, University of Georgia, Athens, GA USA; 3 Department of Microbiology and Immunology, Faculty of Tropical Medicine, Mahidol University, Bangkok, Thailand; 4 Mahidol-Oxford Tropical Medicine Research Unit, Faculty of Tropical Medicine, Mahidol University, Bangkok, Thailand

**Keywords:** Diseases, Immunology, Microbiology

## Abstract

*Burkholderia pseudomallei*, the etiologic agent of melioidosis, is a Gram-negative bacterial pathogen that causes severe disease in humans and animals. In this study, we evaluated the safety and immunogenicity of our lead melioidosis subunit vaccine candidate in cynomolgus macaques. To accomplish this, the 6-deoxyheptan capsular polysaccharide (CPS) from *Burkholderia thailandensis* E555 was purified using a phenol-free extraction process and then conjugated to CRM197 to generate CPS-CRM197. Highly purified, His-tagless *B. pseudomallei* Hcp1 (Hcp1-TL) was also produced. Animals immunized with CPS-CRM197 combined with Hcp1-TL and adjuvanted with Alhydrogel plus CpG DNA (ODN 2006) developed robust CPS-specific IgG and opsonizing antibody responses, alongside strong Hcp1-specific IgG and measurable IFN-γ-secreting T-cell responses. Importantly, the vaccine formulations tested were well tolerated, with no adverse events after three doses. Collectively, these studies establish good manufacturing practices-compatible processes for our vaccine antigens and demonstrate the safety and immunogenicity of the subunit vaccine in non-human primates, supporting continued advancement towards a human clinical trial.

## Introduction

*Burkholderia pseudomallei* is a Gram-negative pathogen that causes melioidosis in humans and animals. The bacterium is an environmental saprophyte that is found in soil and water in endemic areas such as South Asia, Southeast Asia, and Northern Australia, and is predicted to be ubiquitous throughout tropical regions, including Western sub-Saharan Africa, southern Africa, Central and South America, and the Middle East^1–4^. Melioidosis is a multifaceted disease with clinical manifestations that can range from localized skin abscesses to severe pneumonia and septicemia, with the most common presentation being pneumonia^3,5^. *B. pseudomallei* infections are typically acquired through percutaneous inoculation, inhalation, and ingestion^2,3^. In 2016, the burden of human disease was estimated to be ~165,000 melioidosis cases worldwide, with ~89,000 deaths per year^4^. Melioidosis is associated with several comorbidities, and ~80% of melioidosis patients have one or more risk factors such as diabetes mellitus, heavy alcohol use, chronic kidney disease, or thalassemia^3,6^. Given the varied clinical presentations of melioidosis, diagnosis can be challenging, which can result in delayed administration of effective treatment, poor outcomes, and high mortality rates^1–3^. In addition, *B. pseudomallei* is currently classified as a Tier 1 select agent by the U.S. Centers for Disease Control and Prevention (CDC) and is considered a potential biothreat agent^7–9^.

*B. pseudomallei* is a facultative-intracellular pathogen that expresses numerous virulence factors, including both cell-associated and secreted antigens. Important virulence-associated cell-surface-expressed polysaccharides include a 6-deoxyheptan capsular polysaccharide (CPS) and lipopolysaccharide (LPS), which inhibit complement deposition and confer resistance to complement-mediated killing, respectively^10–12^. To enable its intracellular lifestyle, *B. pseudomallei* expresses several specialized secretion systems, including the cluster 3 type III secretion system (T3SS-3) and the cluster 1 type VI secretion system (T6SS-1), both of which promote survival and growth within phagocytic and non-phagocytic cells and are required for full virulence in animal models of infection^13–15^. The intracellular nature of *B. pseudomallei* infections, along with the bacterium’s intrinsic resistance to a wide range of antibiotics, can make eradication difficult. Even with prolonged treatment regimens that include 2–8 weeks of intensive phase therapy followed by 3–6 months of eradication phase therapy, relapse can occur^2,16–18^.

Given the global burden of disease and the potential for *B. pseudomallei* to be misused for malevolent purposes, melioidosis vaccines are urgently needed for both public health and biodefense applications. A variety of vaccine approaches are being pursued to provide complete protection and sterilizing immunity against melioidosis^19^. These include live-attenuated vaccines, glycoconjugate subunit vaccines, outer-membrane vesicle (OMV) vaccines, nanoparticle vaccines, DNA and virus-like particle vaccines^2,20–29^. Subunit-based vaccines are considered a promising approach due to their compatibility with good manufacturing practices (GMP)-compliant antigen production and their favorable safety profiles compared to other vaccine platforms^3,30^. At present, work in our lab is focused on developing glycoconjugate-based subunit vaccines to generate safe and immunogenic candidates that can be used for immunization against melioidosis^21,23,31,32^.

Two promising antigens for use in melioidosis vaccines are the 6-deoxyheptan CPS and hemolysin coregulated protein 1 (Hcp1), which is a component of T6SS-1^15,21,31^. We have previously shown that a CPS-based glycoconjugate subunit vaccine composed of CPS conjugated to diphtheria toxin mutant CRM197 (CPS-CRM197) plus Hcp1 formulated with Alhydrogel and CpG DNA (ODN 2006) stimulates strong antibody responses against both CPS and Hcp1, and robust interferon (IFN)-γ secreting T cell responses against Hcp1 in mice^21^. CPS-specific antibodies were found to be opsonizing and to enhance bacterial uptake into RAW 264.7 cells compared with controls^21,33^. The CPS-CRM197 plus Hcp1 formulation protected 100% of immunized C57BL/6 mice following an aerosol challenge with *B. pseudomallei*, and 70% of the survivors had no culturable bacteria in their lungs, livers, or spleens^21^. More recently, we have shown that this protection is reproducible^34^.

In the present study, we evaluated the safety and immunogenicity of this subunit vaccine in cynomolgus macaques using two different dose levels. The low-dose included 5 µg of CPS as a conjugate, 10 µg of His-tagless Hcp1 (Hcp1-TL), 250 µg of Alhydrogel, and 10 µg of ODN 2006, while the high-dose contained 10 µg of CPS as a conjugate, 20 µg Hcp1-TL, 500 µg of Alhydrogel, and 20 µg ODN 2006. Cynomolgus macaques were selected as a relevant NHP model due to their demonstrated susceptibility to *B. pseudomallei* infection. Previous studies have shown that melioidosis occurs naturally in cynomolgus macaques and can result in systemic infections with clinical manifestations similar to those observed in humans, supporting their utility as a translational model for evaluating vaccine candidates^35^. The low- and high-dose formulations were selected based on prior immunogenicity studies in mice and align with the glycoconjugate and protein antigen quantities described in product monographs for licensed human vaccines, such as PREVNAR 20 (Pfizer) and DAPTACEL (Sanofi Pasteur)^21,36,37^.

GMP-compatible processes were developed for the purification of the 6-deoxyheptan CPS from *Burkholderia thailandensis* and the production of Hcp1-TL from *Escherichia coli*. Cynomolgus macaques were immunized with each of the low- and high-dose formulations, and immune responses to the vaccine antigens were assessed using enzyme-linked immunosorbent assays (ELISA), THP-1 cell-based opsonophagocytosis (OPC) assays, enzyme-linked immunosorbent spot (ELISpot) assays, and ex vivo whole blood stimulation assays. Results demonstrated that strong humoral and measurable cellular immune responses were generated against CPS-CRM197 and Hcp1-TL, with no adverse reactions observed in immunized animals. The results of this study indicate that our melioidosis subunit vaccine is safe and immunogenic in non-human primates (NHPs), supporting its continued advancement towards a human clinical trial.

## Results

### Purification and characterization of *B. thailandensis* CPS antigens

**Fig. 1:**
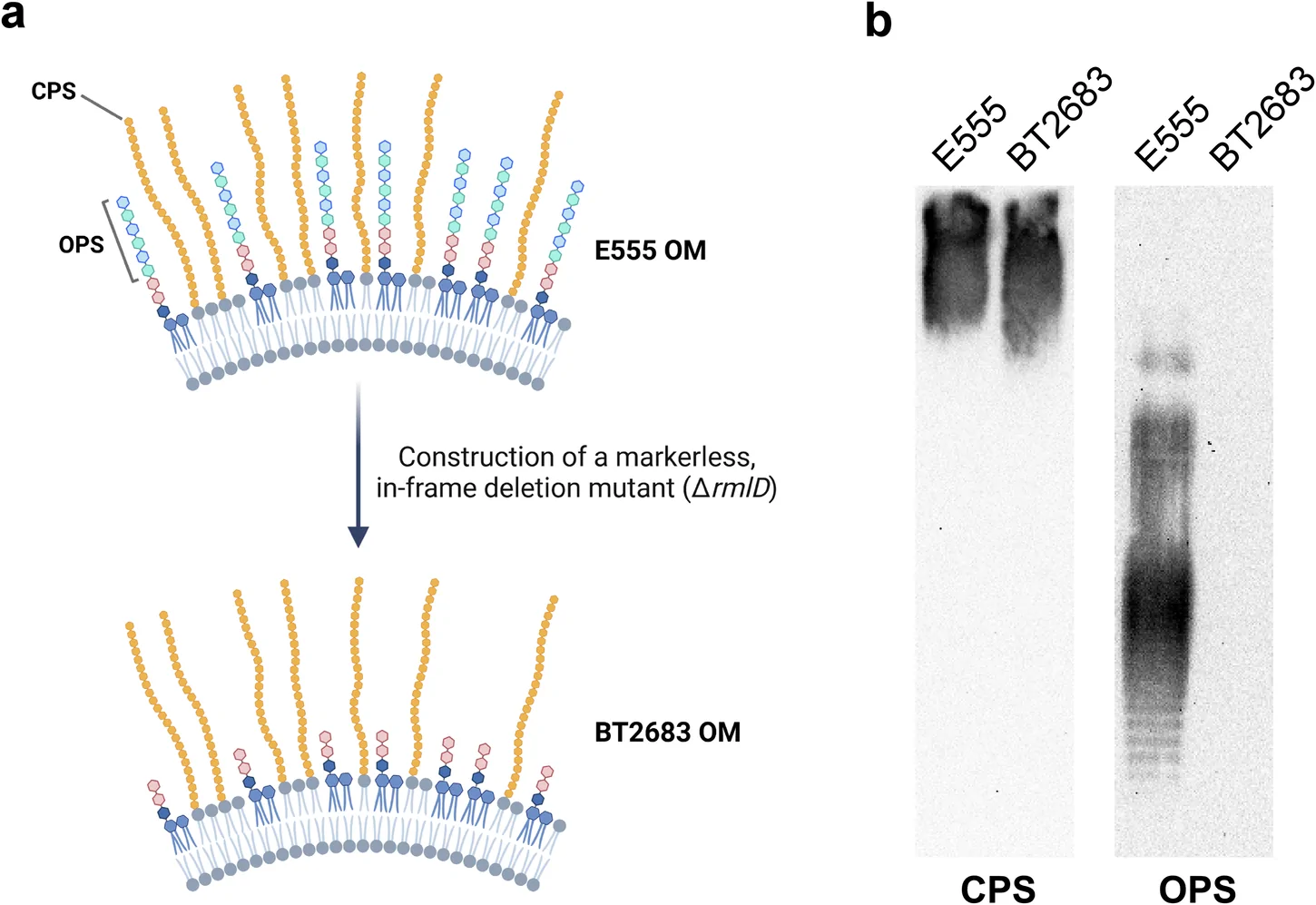
Construction and phenotypic analysis of *B. thailandensis* BT2683 (Δ*rmlD*; OPS mutant). **a** *sacB*-based allelic exchange mutagenesis was used to construct a markerless, in-frame deletion in the OPS biosynthesis operon of *B. thailandensis* E555. The resulting strain, *B. thailandensis* BT2683 (Δ*rmlD*), does not express the OPS antigen. **b** Western immunoblotting was used to confirm loss of OPS expression in *B. thailandensis* BT2683. CPS was detected using mAb 3C5. OPS was detected using mAb Pp-PS-W. Panel (**a**) was created in BioRender. Sengyee, S. (2026) https://BioRender.com/1oifeos.

**Fig. 2:**
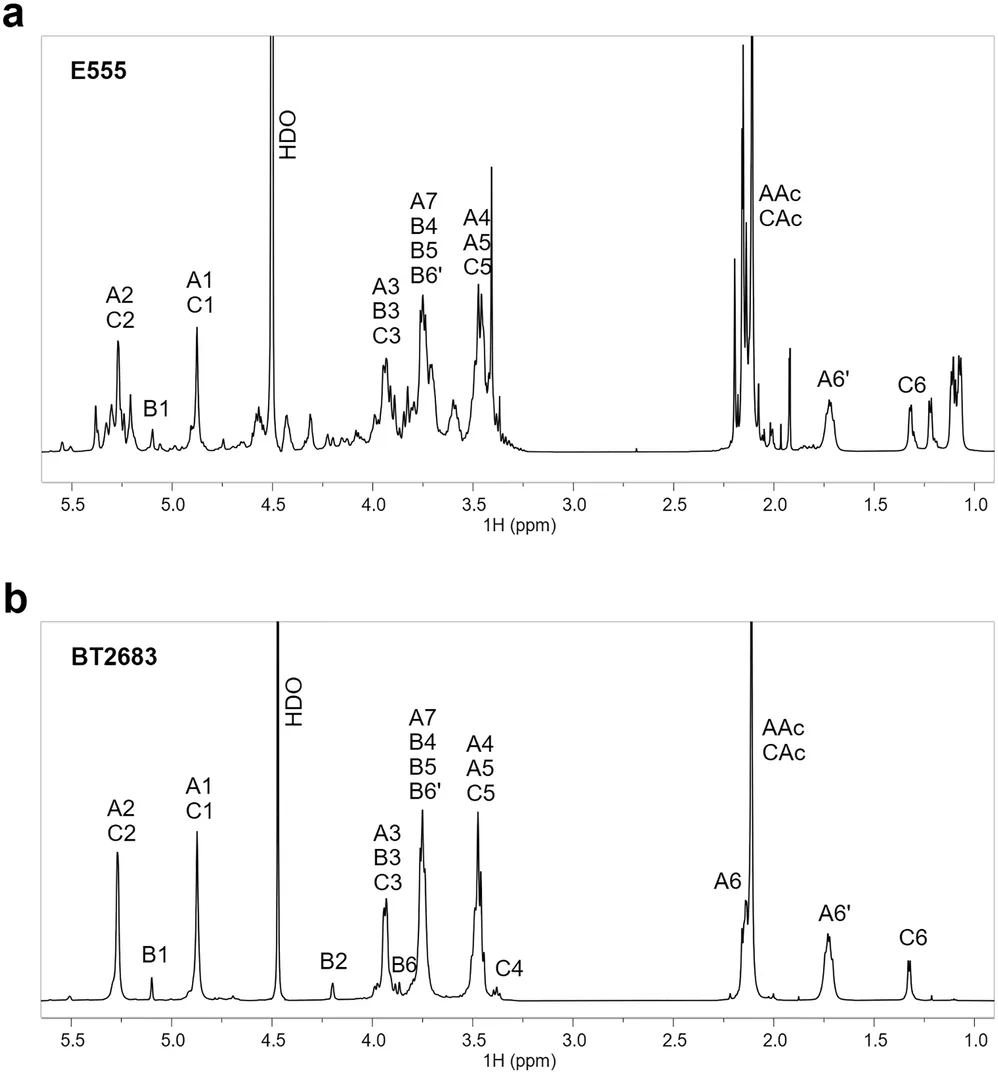
^1^H-NMR analysis of ethanol extracted *B*. *thailandensis* CPS antigens. **a** *B. thailandensis* E555 CPS and **b** *B. thailandensis* BT2683 CPS.

To facilitate the synthesis of the glycoconjugate material used in this study, *B. thailandensis* strain E555 was chosen as a safe, cost-effective source of the CPS antigen. Allelic exchange mutagenesis was used to construct an O-polysaccharide (OPS)-deficient mutant in *B. thailandensis* E555, and this strain was designated *B. thailandensis* BT2683 (Fig. 1a). Western immunoblotting confirmed loss of OPS expression in *B. thailandensis* BT2683 (Fig. 1b). An ethanol extraction procedure was combined with size-exclusion chromatography (SEC) to isolate and purify the CPS from *B. thailandensis* E555 and BT2683. The resulting antigen preparations were free of detectable protein or nucleic acid contamination, as determined by the bicinchoninic acid (BCA) assay or UV spectroscopy, respectively. To assess the structural integrity and homogeneity of the CPS preparations, samples were analyzed by NMR spectroscopy. Using this approach, ^1^H NMR analyses confirmed that the main component of these preparations was the desired 6-deoxyheptan homopolymer (Fig. 2 and Table 1). As expected, the BT2683 sample exhibited a simpler spectrum than E555 because it lacked contaminating OPS signals. In addition to the 6-deoxyheptan, the BT2683 sample contained an α-mannan, as described previously^38^, as well as newly-identified 3-substituted (2-OAc)-β-Rha residues (Suppl. Table 1, Suppl. Fig. 1).

**Table 1:** Assignment of ^1^H-NMR signals of *B. thailandensis* BT2683 CPS

Letter	Residue	Atom chemical shift (ppm)							
		1	2	3	4	5	6	7	OAc
A	→3-(2-OAc)-6d-β-*man*Hep-1→	4.90	5.30	3.96	3.49	3.49	1.75 2.16	3.78	2.13
B	→3-α-Man-1→	5.12	4.22	4.00	3.77	3.83	3.76 3.90		
C	→3-(2-OAc)-β-Rha-1→	4.90	5.30	3.95	3.41	3.51	1.35		2.13

### Synthesis of *B. thailandensis* CPS-CRM197 glycoconjugates

**Fig. 3:**
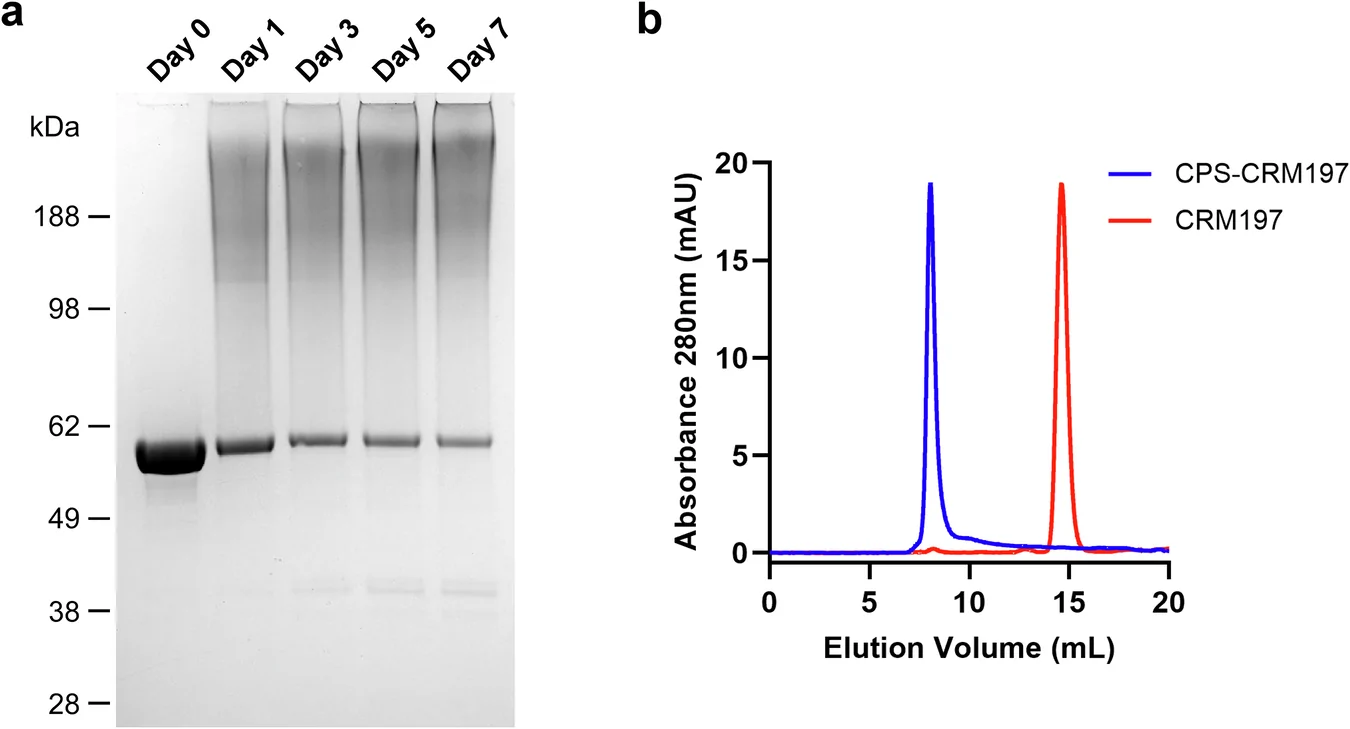
Physical analysis of CPS-CRM197 glycoconjugates. *B. thailandensis* BT2683 CPS was reacted with CRM197 at a 2:1 (w/w) ratio in borate buffer for 7 days. **a** SDS-PAGE and SimplyBlue staining were used to confirm the covalent linkage of CPS to CRM197. Samples were drawn from the reaction mixtures on days 0, 1, 3, and 7. Day 0 represents unconjugated controls. All lanes were loaded with equal amounts of protein to facilitate direct comparisons. Approximately 6 µg of protein was loaded per lane. The positions of protein molecular weight standards are indicated on the left. **b** FPLC-SEC was used to characterize the elution profile of the final glycoconjugate material. ~80 μg of CRM197 (as a conjugate) or unconjugated CRM197 (control) in BupH PBS were loaded onto a Superdex 200 Increase 10/300 GL high-resolution gel filtration column and eluted using BupH PBS at a flow rate of 0.75 mL/min. CRM197 was detected at 280 nm.

The glycoconjugate material used in this study was prepared using CPS isolated from *B. thailandensis* BT2683. The purified CPS was oxidized and covalently linked to CRM197 via reductive amination to produce CPS-CRM197. SDS-PAGE and Coomassie Blue staining were used to monitor conjugation over time and confirmed the coupling of CPS to CRM197 (Fig. 3a). As indicated by shifts in molecular weights of the glycoconjugate material relative to the CRM197 control, CPS was found to couple to the protein carrier in a time-dependent manner. As expected, control reactions containing CRM197 alone or oxidized-CPS alone did not produce high-molecular-weight “smears” when analyzed by SDS-PAGE, confirming that these antigens do not self-conjugate (data not shown). Analysis of the final glycoconjugate material revealed that it contained ~63% (wt/wt) CPS and ~40 endotoxin units (EU)/mg as determined by BCA and limulus amebocyte lysate (LAL) assays, respectively (Suppl. Fig. 2). Fast protein liquid chromatography (FPLC)-SEC analysis also confirmed covalent linkage of CPS to CRM197, as evidenced by the high molecular weight species (CPS-CRM197) eluting at ~8 mL compared to the unconjugated CRM197 control, which eluted at ~15 mL (Fig. 3b). Across nine independent conjugation reactions, final yields averaged ~79% relative to the starting material input (Suppl. Table 2).

### Production of recombinant *B. mallei* Hcp1-TL

**Fig. 4:**
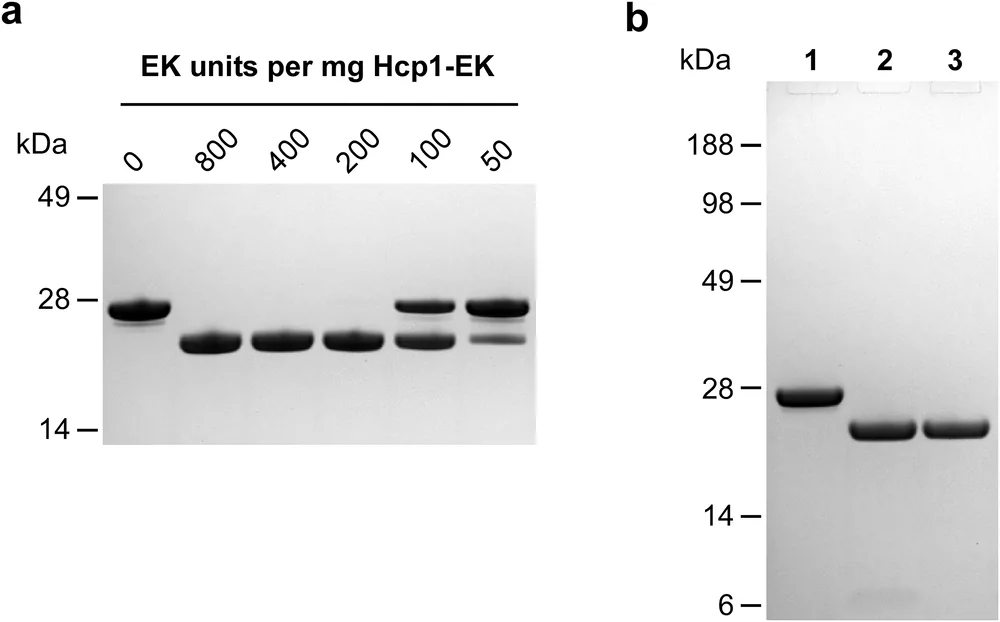
SDS-PAGE analysis of recombinant Hcp1-EK and Hcp1-TL. **a** Hcp1-EK was purified from *E. coli* lysates using cobalt chromatography, and the N-terminal His-tag was removed via enterokinase (EK) cleavage. The protein was incubated with EK at various concentrations (0–800 U per mg of protein) at 37 °C for 20 h to determine the optimal concentration for efficient cleavage. **b** Cleavage of Hcp1-EK using 200 U of EK and assessment of the purity of the resulting Hcp1-TL following cobalt chromatography. Hcp1-EK (lane 1), Hcp1-EK incubated with EK for 20 h (lane 2), and Hcp1-TL (lane 3). SDS-PAGE on 12% Bolt gels and SimplyBlue staining were used to visualize the proteins. Approximately 3 µg of protein was loaded per lane. Positions of the molecular weight standards (kilodaltons) are indicated on the left.

To obtain Hcp1-TL for use in this study, a recombinant version of the protein with an enterokinase cleavable N-terminal His-Tag (Hcp1-EK) was expressed in *E. coli*. Hcp1-EK was extracted from whole-cell pellets in soluble form and purified to homogeneity using cobalt affinity chromatography. The His-tag was removed from Hcp1-EK by enterokinase cleavage, and the resulting protein, Hcp1-TL, was then isolated using cobalt resin. The optimal enterokinase concentration for removing the His-tag was determined by incubating the protein with varying enzyme concentrations and assessing cleavage by SDS-PAGE (Fig. 4a). Based on these results, 200 units of enterokinase per milligram of Hcp1-EK were used to obtain Hcp1-TL for these studies. SDS-PAGE was used to confirm the removal of the His-tag from Hcp1-EK and to assess the purity and structural integrity of Hcp1-TL (Fig. 4b). Endotoxin concentrations in the Hcp1-TL preparations were < 1 EU/mg, as determined by LAL assay (Suppl. Fig. 2).

### Analysis of antibody responses raised against CPS, Hcp1-TL, and CRM197

**Fig. 5:**
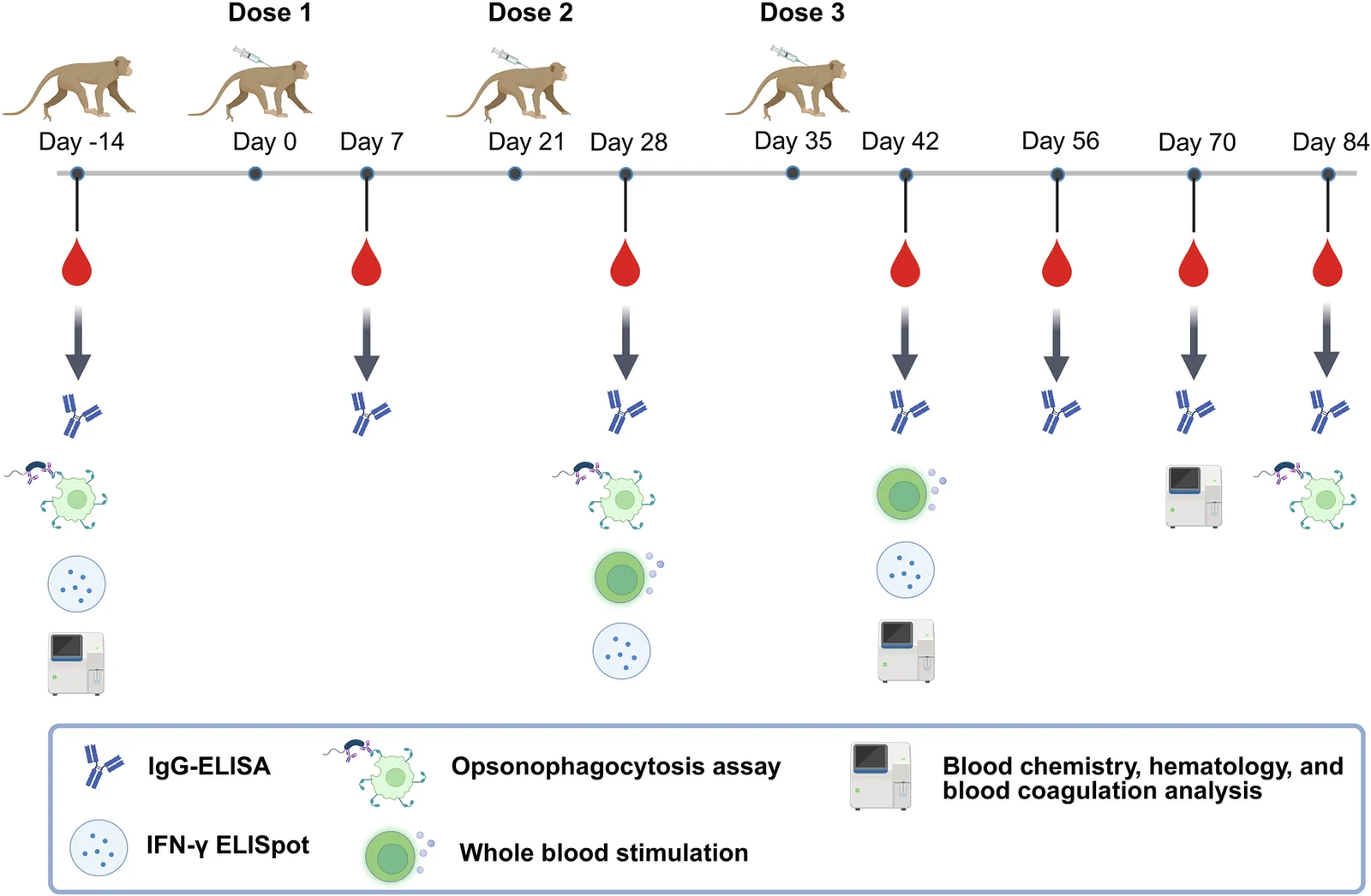
Study design. Cynomolgus macaques (*n* = 4 per group) were immunized s.c. with the low-dose formulation (5 µg of CPS/dose as a conjugate, 10 µg Hcp1/dose, 10 µg ODN 2006/dose, 250 µg of Alhydrogel/dose) or the high-dose formulation (10 µg of CPS/dose as a conjugate, 20 µg Hcp1/dose, 20 µg ODN 2006/dose, 500 µg of Alhydrogel/dose) on days 0, 21, and 35. Humoral and cellular immune responses against the vaccine antigens were determined using IgG ELISA, opsonophagocytosis assays, IFN-γ ELISpot, and whole blood stimulation assays, as indicated in the timeline. Blood chemistry, hematology, and blood coagulation analysis were performed on days -14, 42, and 70. Created in BioRender. Sengyee, S. (2026) https://BioRender.com/k8l9ib6.

To examine the immunogenicity of the CPS-CRM197 and Hcp1-TL antigens, cynomolgus macaques were immunized three times with low- and high-dose formulations in two separate experiments. In each study, two male and two female animals were administered the vaccine antigens formulated with Alhydrogel and ODN 2006 via subcutaneous (s.c.) injections on days 0, 21, and 35, as outlined in Fig. 5. To assess antibody responses, plasma samples were collected two weeks before the first immunization and then on days 7, 28, 42, 56, 70, and 84 (Fig. 5). Antigen-specific IgG titers were determined by ELISA at each time point.

**Fig. 6:**
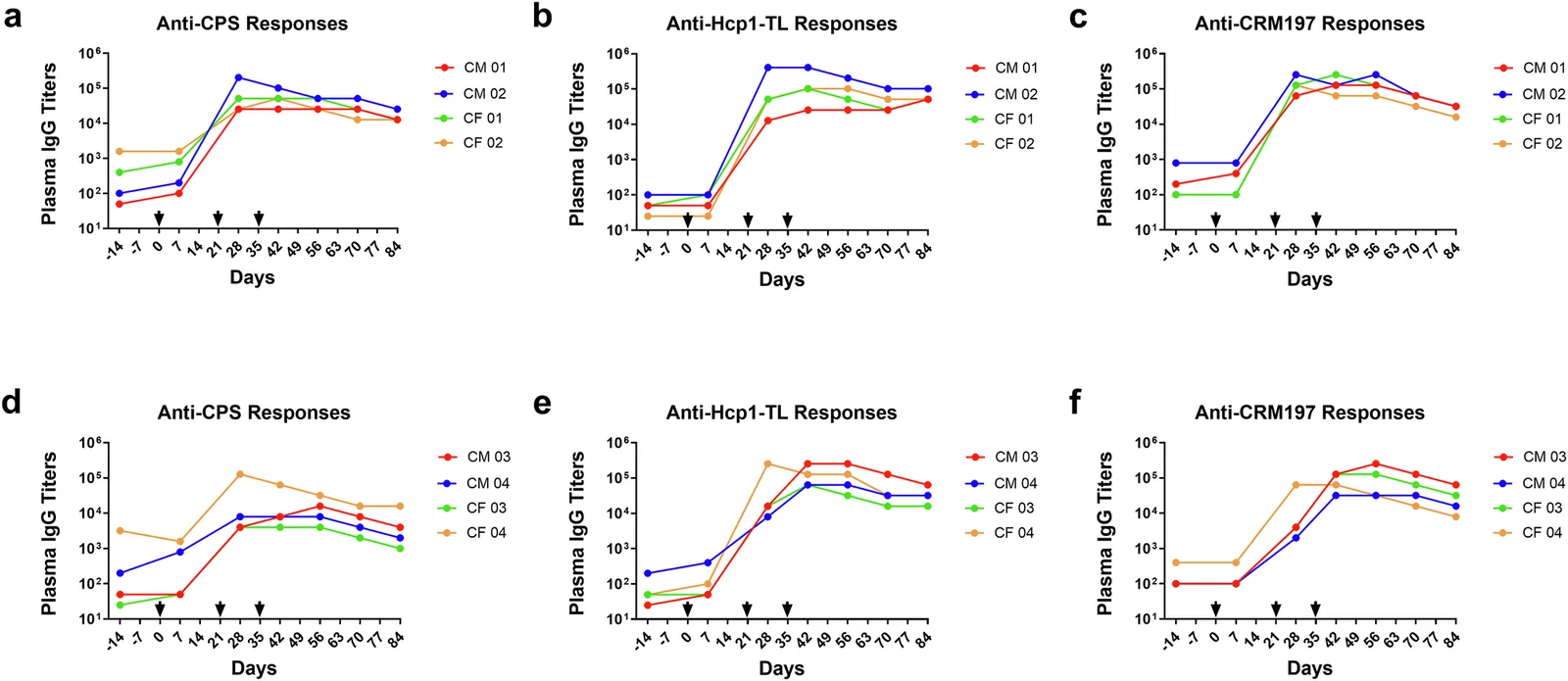
Characterization of antibody titers raised against CPS, Hcp1-TL and CRM197. Cynomolgus macaques (*n* = 4 per group) were immunized s.c. on days 0, 21, and 35 with CPS-CRM197 plus Hcp1-TL. Plasma samples were collected two weeks before immunization (day -14) and on days 7, 28, 42, 56, 70, and 84 post-immunization to determine IgG responses using ELISA. **a**–**c** IgG titers against CPS, Hcp1-TL, and CRM197, respectively, in cynomolgus macaques immunized with low-dose formulation. **d**–**f** IgG titers against CPS, Hcp1-TL, and CRM197, respectively, in cynomolgus macaques immunized with high-dose formulation. Plasma IgG titers are plotted for each animal. The black arrows indicate the days of immunization. CM cynomolgus macaque male, CF, cynomolgus macaque female.

In the low-dose group, all animals exhibited high-titer total IgG responses (endpoint titers > 10^4^) against CPS, Hcp1-TL, and CRM197 by day 28 (Fig. 6a–c). The IgG endpoint titers showed marked increases compared to pre-immune levels, with fold increases ranging from 16-fold to 2048-fold for CPS, 256-fold to 4096-fold for Hcp1-TL, and 320-fold to 1280-fold for CRM197. In addition, the IgG titers remained high (endpoint titers > 10^4^) against all antigens through day 84 (Fig. 6a–c). Similar results were observed in the high-dose group, with all animals exhibiting high-titer total IgG responses (endpoint titers > 10^3^) against CPS, Hcp1-TL, and CRM197 by day 28 (Fig. 6d–f). Compared with pre-immune plasma, IgG endpoint titers increased 40-fold to 160-fold for CPS, 40-fold to 5120-fold for Hcp1-TL, and 20-fold to 160-fold for CRM197. As in the low-dose group, IgG titers in the high-dose group remained high (endpoint titers > 10^3^) against all antigens through day 84 (Fig. 6d–f). Interestingly, animals that received the high-dose formulation showed slightly lower overall IgG responses at day 28 than those in the low-dose group. Taken together, these results demonstrate that both the low- and high-dose formulations elicited high-titer antigen-specific IgG responses in NHPs.

**Fig. 7:**
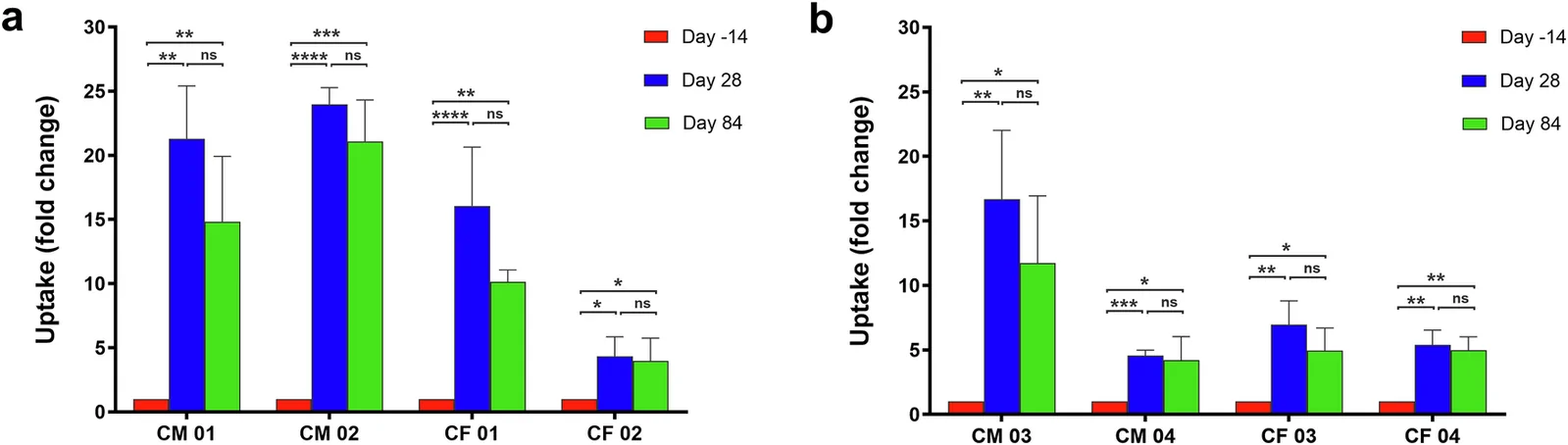
Opsonophagocytic activity of immune serum samples. Functional analysis of antibody responses in cynomolgus macaques following s.c. immunizations on days 0, 21, and 35 with (**a**) low-dose and (**b**) high-dose formulations. *B. thailandensis* E555 was incubated with heat-inactivated pre-immune plasma (day -14) and immune plasma on day 28 and day 84. After 1 h of incubation, opsonized bacteria were added to THP-1-derived macrophage cells at an MOI of 0.1. Bacterial uptake was quantitated at 3 h post-infection. Values represent the fold change in uptake of immune plasma (day 28 and day 84) compared to baseline (day -14). Mean ± standard deviations of three independent experiments conducted in duplicate are illustrated. CM cynomolgus macaque male, CF cynomolgus macaque female, ns not significant; *P < 0.05; **P < 0.01; ***P < 0.001; ****P < 0.0001.

To evaluate the functionality of the CPS-specific antibodies generated by the vaccine formulations, OPC assays were performed using THP-1-derived macrophages. Before conducting the assays, phorbol 12-myristate 13-acetate (PMA)-differentiated THP-1 cells were confirmed to have increased levels of macrophage markers by flow cytometry (Suppl. Fig. 3). *B. thailandensis* E555, a non-pathogenic species that expresses the same 6-deoxyheptan CPS as *B. pseudomallei*, but a different T6SS-1 Hcp1, was used as a surrogate for *B. pseudomallei*, which enabled assessment of the opsonizing potential of the CPS-specific antibodies, but not Hcp1-specific antibodies. As shown in Fig. 7, pre-incubation of *B. thailandensis* E555 with 5% heat-inactivated (HI) plasma collected from animals on days 28 and 84 in both the low- and high-dose groups significantly enhanced bacterial uptake compared to pre-immune plasma. For animals immunized with the low-dose formulation, the mean fold change increase in bacterial uptake was 16-fold on day 28 and 12-fold on day 84 (Fig. 7a). In contrast, animals receiving the high-dose formulation exhibited a mean fold change of 8 on day 28 and 6 on day 84, indicating reduced opsonophagocytic activity compared to the low-dose vaccine. No significant differences in bacterial uptake were observed between immune plasma samples collected on day 28 and day 84 for either the low- or high-dose vaccine formulation. Notably, variability in fold changes in bacterial uptake was observed among animals, which correlated with their background antibody levels; animals with higher pre-existing CPS antibodies showed lower fold changes in bacterial uptake after immunization. Collectively, these results show that following immunization of NHPs, the CPS-CRM197 plus Hcp1-TL vaccine can generate antibodies with opsonizing activity.

### Characterization of IFN-γ-secreting T cell responses against Hcp1-TL and CPS-CRM197

**Fig. 8:**
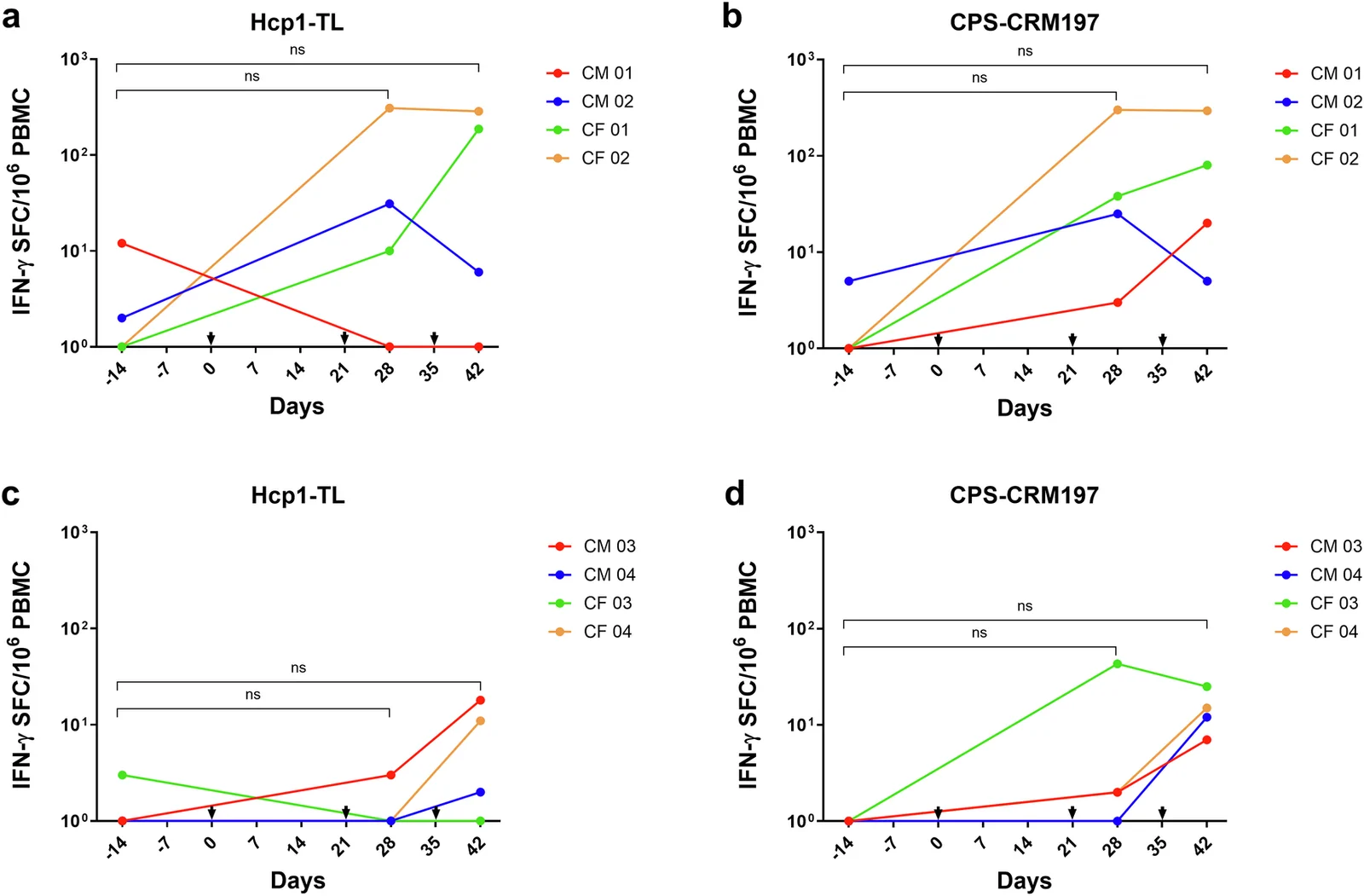
Characterization of cellular immune responses raised against Hcp1-TL and CPS-CRM197. Cynomolgus macaques (*n* = 4 per group) were immunized s.c. on days 0, 21, and 35 with CPS-CRM197 plus Hcp1-TL. PBMCs were isolated from whole blood 2 weeks before the first dose (day -14), 1 week after the second dose (day 28), and 3 weeks after the third dose (day 42). IFN-γ-secreting T cell responses against Hcp1-TL and CPS-CRM197 were assessed using IFN-γ ELISpot assay. **a**, **b** IFN-γ-secreting T cell responses against Hcp1-TL and CPS-CRM197, respectively, in cynomolgus macaques immunized with low-dose formulation. **c**, **d** IFN-γ-secreting T cell responses against Hcp1-TL and CPS-CRM197, respectively, in cynomolgus macaques immunized with high-dose formulation. Each dot represents the mean results of assays performed in duplicate for each animal. The black arrows indicate the days of immunization. SFC spot-forming cell, CM cynomolgus macaque male; CF cynomolgus macaque female. ns, not significant.

To assess the cellular immune responses to the subunit vaccine antigens, peripheral blood mononuclear cells (PBMCs) isolated from whole blood from all immunized animals two weeks before the first dose (day -14), one week after the second dose (day 28), and third dose (day 42) were re-stimulated with Hcp1-TL and CPS-CRM197. ELISpot assays were then used to analyze IFN-γ-secreting T cell responses. Phytohaemagglutinin (PHA) was included as a positive control to ensure T cell responsiveness (data not shown). IFN-γ-secreting T cell responses were detected following immunization with the low- and high-dose formulations (Fig. 8). The median levels of IFN-γ responses at day -14, day 28, and day 42 in the low-dose group were as follows: Hcp1-TL 1.5, 20.5, and 96.5 SFC/10^6^ PBMC, and CPS-CRM197 1, 31.5, and 50 SFC/10^6^ PBMC, respectively. The median levels of IFN-γ responses at day -14, day 28, and day 42 in the high-dose group were as follows: Hcp1-TL 1, 1, and 6.5 SFC/10^6^ PBMC, and CPS-CRM197 1, 2, and 13.5 SFC/10^6^ PBMC, respectively. Results showed that the low-dose formulation consistently elicited higher IFN-γ-secreting T cell responses to both Hcp1-TL and CPS-CRM197 at day 28 and day 42 compared with the high-dose formulation. Collectively, these findings demonstrate that our subunit vaccine can induce antigen-specific IFN-γ-secreting T cell responses in NHPs.

### Analysis of IFN-γ cytokine responses against Hcp1-TL and CPS-CRM197

To further evaluate the immune responses elicited by our subunit vaccine formulations, we measured IFN-γ levels after ex vivo whole blood stimulation with Hcp1-TL and CPS-CRM197. Whole blood samples collected one week after the second dose (day 28) and one week after the third dose (day 42) were stimulated for 48 h with Hcp1-TL or CPS-CRM197, and IFN-γ responses were quantified by ELISA. Higher IFN-γ responses were observed against both Hcp1-TL and CPS-CRM197 at day 28 and day 42 following immunization with the low-dose formulation (Suppl. Fig. 4a, b) compared to the group that was immunized with the high-dose formulation (Suppl. Fig. 4c, d). The median levels of IFN-γ following immunization with the low-dose formulation were 175.4 pg/ml and 62.7 pg/ml for Hcp1-TL, and 420.9 pg/ml and 168.5 pg/ml for CPS-CRM197 on days 28 and 42, respectively. The high-dose formulation elicited substantially lower IFN-γ responses than the low-dose formulation, with median IFN-γ levels of 3.4 pg/ml and 7.3 pg/ml for Hcp1-TL and 9.3 pg/ml and 16.3 pg/ml for CPS-CRM197 on days 28 and 42, respectively. Taken together, these findings indicate that the low-dose formulation induced stronger antigen-specific IFN-γ responses than the high-dose formulation in NHPs following immunization.

### Assessment of subunit vaccine safety in cynomolgus macaques

To assess the safety of the vaccine formulations, blood samples were collected at three time points for clinical chemistry analysis, as outlined in Fig. 5. The individual body weights of each animal were measured weekly throughout the study. Blood clinical chemistry, hematology, and coagulation measurements were performed on samples obtained from individual animals two weeks before the first immunization (day -14), one week after the third dose (day 42), and three weeks after the third dose (day 70). All measurement parameters for the animals immunized with the low-dose formulation are shown in Supplementary Tables 3, 4, and 5. There were no test material-related clinical observations or changes in body weight, qualitative food consumption, hematology, coagulation, or clinical chemistry parameters following immunization with the low-dose formulation. All measurement parameters for the animals immunized with the high-dose formulation are shown in Supplementary Tables 6, 7, and 8. There were no test material-related clinical observations or changes in body weight, qualitative food consumption, hematology parameters, or clinical chemistry parameters following immunization with the high-dose formulation. There were minimal changes observed in the coagulation parameters with mild prolongations in the activated partial thromboplastin time (APTT) on day 42 in three of the animals in the high-dose group (cynomolgus macaque male CM 04 and cynomolgus macaque females CF 03 and CF 04). By day 70, however, the values had returned to levels comparable to the baseline values observed on day -14 (Supplementary Table 6). Both the low- and high-dose formulations were well tolerated by all animals, with no adverse effects from immunization or blood sampling.

## Discussion

*B. pseudomallei* is an emerging public health threat that causes a significant amount of morbidity and mortality in endemic regions. The global distribution of this important pathogen is broader than previously recognized, and no vaccines are currently available to protect humans and animals against melioidosis^4,20,39^. The development of subunit vaccines for immunization against melioidosis is a promising approach that offers safety and stability advantages over some other vaccine platforms. Additionally, this strategy enables the rational design of antigenically defined vaccine formulations comprising protective antigens and adjuvants that can promote specific immune responses^20,21,31,33,34,40^. This is particularly important for combating disease caused by facultative intracellular pathogens such as *B. pseudomallei*, since an effective vaccine will likely require stimulation of both humoral and cellular immune responses to control infections. Several studies from our lab and others have shown that vaccines consisting of CPS-based glycoconjugates combined with purified *Burkholderia* proteins stimulate robust antigen-specific antibody and T cell responses that provide high levels of protection against inhalational challenges of *B. pseudomallei* in mice^21,23,33^. In the present study, we tested our lead subunit vaccine candidate, CPS-CRM197 plus Hcp1-TL formulated with Alhydrogel and ODN 2006, at two different dose levels in both male and female cynomolgus macaques. Whether immunized with a low- or high-dose formulation, all animals exhibited robust antigen-specific IgG responses against CPS and Hcp1-TL, as well as measurable Hcp1-specific IFN-γ responses. Importantly, no adverse effects were observed following immunization with either formulation.

CPS is an important protective antigen and a key component of various melioidosis vaccine candidates^21,31,33,41^. Having a source from which to safely isolate this polysaccharide and an efficient extraction procedure to obtain highly pure material will be critical for advancing the development of CPS-containing vaccines to meet eventual GMP-compatible requirements. To address these considerations, we 1) utilized *B. thailandensis* E555 as a non-pathogenic source of CPS, 2) genetically engineered the strain to be devoid of OPS, and 3) developed an ethanol-based extraction procedure to simplify the isolation of high-purity CPS. The CPS biosynthetic gene clusters in *B. thailandensis* E555 and *B. pseudomallei* K96243 are highly similar, and NMR spectroscopy has shown that *B. thailandensis* E555 produces a structurally identical 6-deoxyheptan CPS^42,43^. To eliminate the possibility of OPS contamination in our preparations, an OPS-deficient derivative of E555 (strain BT2683) was constructed and used for CPS purification^42^. For more than three decades, the standard method for extracting CPS from *B. pseudomallei* and *B. thailandensis* has relied on the use of hot phenol, which is both cumbersome and presents potential safety issues^21,23,32,33,38,41,44–46^. To simplify this process and improve safety, we developed an ethanol-based extraction procedure that yielded high-quality CPS preparations. NMR results demonstrated that the CPS purified from *B. thailandensis* BT2683 using this method was structurally identical to CPS expressed by *B. thailandensis* E555 and was of higher purity. Establishment of this methodology, in combination with the use of a non-pathogenic source of CPS, will enable the safe, efficient, and cost-effective purification procedures for the future production of this important vaccine antigen under GMP conditions.

Hcp1 is a highly immunogenic protective antigen that is a structural component of T6SS-1 expressed by *B. pseudomallei* and *B. mallei*^15,21,34^. Past studies examining the protective capacity of this protein have used recombinant versions of Hcp1 harboring either N- or C-terminal His-tags^15,21^. Here, we prepared recombinant Hcp1-EK to facilitate the production of Hcp1-TL for use in our subunit vaccine formulations. His-tag-free proteins are preferred for use in humans since these tags have been reported to negatively affect proteins, with respect to toxicity, changes in their structure and/or biological activity^47–49^. Hcp1-TL has previously been tested in mice in combination with CPS-CRM197, Alhydrogel, and ODN 2006, and is safe, immunogenic, and protective in inhalational models of melioidosis and glanders^34,50^. The methods developed for the purification of Hcp1-TL will be helpful for the future production of this antigen under GMP conditions.

To obtain immunogenicity and safety data in a higher-order species, our vaccine antigens were evaluated in 8 sexually mature cynomolgus macaques. Using plasma samples collected at various time points after immunization of animals with CPS-CRM197 plus Hcp1-TL, formulated with Alhydrogel and ODN 2006, we observed increased IgG levels against CPS, Hcp1-TL, and CRM197. High antigen-specific IgG titers were generated after the second dose and remained high throughout the study (through day 84). Interestingly, the low-dose formulation elicited higher IgG titers against vaccine antigens than the high-dose formulation. Previous studies have shown that lower antigen doses formulated with appropriate adjuvants can elicit humoral and cellular immune responses comparable to those induced by higher-dose regimens^51^. For B cells, increasing the antigen dose does not necessarily enhance antibody responses, as humoral immunity can plateau due to germinal center saturation, antigen availability, and affinity-based selection^52^. For T cells, antigen dose is a key determinant of priming and differentiation, with higher doses associated with the induction of regulatory or functionally constrained responses that may limit effector activity. In contrast, lower antigen doses can favor the generation of more functional and qualitatively superior immune responses^53^. Together, these mechanisms may contribute to the comparable or enhanced humoral and cellular immune responses observed with the low-dose formulation in this study.

Previous studies in C57BL/6 mice immunized with the same vaccine formulation demonstrated high levels of anti-CPS and anti-Hcp1 antibodies following immunization, and these antibodies were associated with protection against a challenge with *B. pseudomallei*^34^. In a previous study, rhesus macaques immunized with *B. pseudomallei* 1026b OMVs elicited high-titer OMV-, LPS-, and CPS-specific IgG responses^54^. This OMV vaccine candidate demonstrated cross-protection against inhalational glanders in both mice and rhesus macaques^55^. Additionally, OMV vaccination has been shown to prevent lung pathology in a rhesus macaque model of melioidosis^27^. Future studies will be necessary to evaluate the protective capacity of our subunit vaccine formulations in an NHP model of melioidosis.

In this study, we performed OPC assays using differentiated THP-1 cells and plasma from the NHPs immunized with our subunit vaccine. The results demonstrated that CPS-specific antibodies were functional, as immune serum collected on days 28 and 84 promoted the uptake of *B. thailandensis* E555 into THP-1 cells. These findings are consistent with previous studies demonstrating that CPS-based glycoconjugate vaccines stimulate CPS-specific opsonizing antibody responses in mice^21,33^. The plasma from the low-dose immunizations was associated with more consistent, higher-fold increases in bacterial uptake. Further studies will be required to understand this interesting phenomenon. While lower fold changes in bacterial uptake were observed in the NHPs with higher baseline levels of anti-CPS IgG before immunization, significantly higher uptake was observed using plasma obtained after 2 or 3 doses of the vaccine formulation. Since the NHPs used in this study were of Asian origin, they may have been exposed to closely related soil bacteria, including non-pathogenic *Burkholderia*, which may account for the higher background levels of CPS-specific IgG detected in some pre-immune plasma samples. Our finding that CPS-specific antibodies generated in the NHPs were functional is promising, given that previous studies have shown that opsonization of *B. pseudomallei* with serum from melioidosis survivors enhanced phagocytosis and killing in human THP-1 cells^56,57^. It is important to note that while CPS-specific antibody responses have been identified in melioidosis patients, with levels differing significantly from those observed in healthy donors, their association with survival or protection remains unclear^58^.

IFN-γ is an important cytokine that promotes Th1-like immune responses, enhances phagocytosis by macrophages, and the clearance of intracellular pathogens^59–61^. Using PBMCs isolated from the NHPs after immunization with 2 or 3 doses of our vaccine formulation, we demonstrated higher IFN-γ-secreting T cell responses than in pre-immune controls. These findings are consistent with previous studies showing that mice immunized with CPS-based glycoconjugate subunit vaccine candidates containing Hcp1 produced robust IFN-γ secreting T cell responses^21,34^. The relatively modest IFN-γ T cell responses observed in this study, may reflect the suboptimal immunostimulatory activity of the ODN 2006 adjuvant in NHPs. Notably, CpG ODNs that are highly active in mice often exhibit reduced potency in primates, suggesting that significantly higher doses of ODN 2006 (10 to 50 times more) may be required to achieve optimal activation of humoral and cellular immune response^62^. Future studies will be needed to optimize adjuvant formulations, route of immunization, and dosing to enhance the immunogenicity of the vaccine antigens.

Previous studies using human PBMC samples from northeastern Thailand reported an association between survival in melioidosis patients and the number of IFN-γ-secreting T cells responding to whole bacteria and purified protein antigens, including Hcp1, AhpC, LolC, OppA, and PotF^63–65^. Likewise, multiple studies indicate that enhanced IFN-γ-secreting T cell responses to *B. pseudomallei* antigens correlate with survival in patients with melioidosis^63–67^. We also observed measurable IFN-γ responses in whole blood from the immunized NHPs upon re-stimulation with our vaccine antigens. These findings are consistent with a previous study in which mice immunized with our CPS-based glycoconjugate subunit vaccine exhibited robust IFN-γ responses against Hcp1 compared with an adjuvant control^34^. Additionally, IFN-γ and IL-22 were increased in immunized mice challenged with *B. pseudomallei* K96243, and these elevated cytokines were associated with higher levels of protection and recovery post-challenge^34^.

Analysis of the blood clinical chemistry, hematology, and coagulation results obtained during the studies indicated that the vaccine formulations were well tolerated by the animals. Although mild changes in the APTT were observed in three out of four NHPs immunized with the high-dose formulation, these were transient and not associated with any clinical signs of illness or other hematological abnormalities. Multiple-dose toxicity studies in NHPs have suggested that the formation of antigen-antibody complexes following repeated immunization may contribute to complement activation, vascular inflammation, and secondary consumptive coagulopathy^68^.

Collectively, our findings indicate that CPS-CRM197 plus Hcp1-TL, formulated with Alhydrogel and ODN 2006, was well tolerated and immunogenic in NHPs. This formulation stimulated antigen-specific humoral and cellular immune responses in NHPs, supporting previous studies demonstrating its potential as a promising vaccine candidate for immunization against melioidosis^21,34^. Moreover, the observed differences between the low- and high-dose formulations suggest that dose optimization may be critical for maximizing vaccine efficacy. Challenge studies will be required to determine the protective efficacy of this vaccine candidate in an NHP model of melioidosis and advancement of this subunit vaccine toward human clinical trials. Importantly, this study provides an improved method for purifying *B. thailandensis* CPS, a procedure for producing His-tagless Hcp1, and methods for assessing immune responses against these antigens in NHPs.

## Methods

### Bacterial strains, growth conditions, and reagents

The bacterial strains used in this study are described in Table 2. *E. coli* and *B. thailandensis* strains were grown at 37 °C on Luria Bertani-Lennox (LBL; Difco) agar or in LBL broth or minimal media (M9TG; 1X M9 Minimal Salts, 0.5% Tryptone, 0.4% glucose, 2 mM MgSO_4_, 0.1 mM CaCl_2_) broth. When appropriate, antibiotics were added at the following concentrations: 100 µg/ml ampicillin, 25 μg/ml kanamycin (Km), or 15 μg/ml polymyxin B (Pm) for *E. coli*, and Km at 500 μg/ml for *B. thailandensis*. Yeast tryptone (YT) broth or YT agar containing 15% sucrose was used in gene-replacement experiments, as described below. Bacterial stocks were maintained at −80 °C as 20% glycerol suspensions. Unless stated otherwise, all chemicals and reagents were purchased from Sigma.

**Table 2:** Bacterial strains, plasmids and primers used in this study

Strains	Description^a^	Reference/source
*Escherichia coli*		
TOP10	Lab strain for cloning and protein expression; Km^S^	Life Technologies
S17-1	Mobilizing strain with transfer genes of RP4 integrated on the chromosome; Km^S^, Pm^S^	^73^
*Burkholderia thailandensis*		
E555	Wild type; soil isolate from Cambodia	^74^
BT2683	OPS-deficient derivative of E555; Δ*rmlD*	This study
**Plasmids**	**Description**^a^	**Reference/source**
pMo130	Suicide vector for allelic exchange in *Burkholderia*; ColE1 *ori* RK2 *oriT xylE sacB*; Km^R^	^69^
pMo-Bt*rmlD*5’	pMo130 harboring a 626 bp fragment containing the 5’ region of *rmlD* (Bt locus tag: BTHE555_RS0101745)	This study
pMo-BtΔ*rmlD*	pMo130 harboring *rmlD* with a 354 bp in-frame deletion	This study
pBAD/HisA	Arabinose inducible, 6xHis-Tag expression vector; Ap^R^	Life Technologies
pBADHisA-Bmhcp1-EK	pBAD/HisA containing *B. mallei hcp1* (BMAA0742) with an N-terminal His-Tag and an enterokinase cleavage site; Ap^R^	This Study
**Primers**^b^	**Sequence (5′-3′)**	**Reference/source**
BtrmlD-5FH	CATGAAGCTTTTCAGGACAATCATTCCCGATCCG	This study
BtrmlD-5RPs	TTGACCACCTTAGCTGCAGCGCCATC	This study
BtrmlD-3FPs	GCGATCGATTGCTGCAGGGACGGCTCAC	This study
BtrmlD-3RXb	CATGTCTAGAGACACATTCGGCGAACAACCCGTG	This study
BtrmlD-F1	GACCGACTATTGGTATCCGGAGTTCG	This study
BtrmlD-R1	TTCGTCATCTGCCAGATGAGCGAAGC	This study
Bmhcp1-FXh	CATGCTCGAGCTGGCCGGAATATATCTCAAGGTC	This study
Bmhcp1-RH	CATGAAGCTTTCAGCCATTCGTCCAGTTTGCGGC	This study

^a^*Ap* ampicillin, *Km* kanamycin, *Pm* polymyxin B, *R* resistant, *s* susceptible.

^b^Restriction sites in the linker regions are underlined.

### Recombinant DNA techniques

DNA manipulations were performed using standard methods. Restriction enzymes and T4 DNA Ligase (New England BioLabs) were used according to the manufacturer’s instructions. PCR was performed using an Expand High Fidelity PCR System (Roche Applied Science) or GoTaq polymerase (Promega). PCR and restriction-digested products were purified using a QIAquick Gel Extraction Kit (Qiagen). Plasmids were purified using a QIAprep Spin Miniprep Kit (Qiagen). Genomic DNA was purified using a Wizard Genomic DNA Purification kit (Promega). Chemically competent *E. coli* TOP10 cells were transformed as per the manufacturer’s instructions (Invitrogen). Oligonucleotide primers were obtained from Integrated DNA Technologies (Coralville, IA). DNA sequencing was performed by ACGT, Inc. (Wheeling, IL). The oligonucleotide primers used in this study are described in Table 2.

### Construction of *B. thailandensis* BT2683

To facilitate the construction of an O-polysaccharide (OPS) deficient mutant in *B. thailandensis* E555, gene replacement was performed using the *sacB*-based allelic exchange vector pMo130 essentially as previously described in ref. ^69^. To construct plasmid pMo-ΔBt*rmlD*, two DNA fragments were PCR amplified from *B. thailandensis* E555 genomic DNA, digested and sequentially cloned into pMo130. First, the BtrmlD-5FH/BtrmlD-5RPs primer pair was used to PCR-amplify a 636-bp DNA fragment that includes the 5’ region of *rmlD* and upstream DNA. This fragment was digested with HindIII and PstI and cloned into similarly digested pMo130, resulting in pMo-BtrmlD5. Next, the BtrmlD-3FPs/BtrmlD-3RXb primer pair was used to PCR-amplify a 671-bp DNA fragment that includes the 3’ region of *rmlD* and downstream DNA. This fragment was digested with PstI and XbaI and cloned into similarly digested pMo130, resulting in pMo-BtΔ*rmlD*.

To construct *B. thailandensis* BT2683 (Δ*rmlD* mutant), *E. coli* S17-1 was used to mobilize pMo-BtΔ*rmlD* into *B. thailandensis* E555 via conjugative mating for 8 h at 37 °C. To select for transconjugants, conjugation mixtures were plated onto LB-Km-Pm agar and incubated for 48 h at 37 °C. To isolate sucrose-resistant colonies, individual transconjugants were grown in YT broth for 4 h at 37 °C and 200 rpm, then spread onto YT media containing 15% sucrose and incubated for 24–48 h at 37 °C. Sucrose-resistant colonies were then screened for Km susceptibility by replica plating. The presence of the mutant allele was verified by PCR using the BtrmlD-F1 and BtrmlD-R1 primer pair.

### CPS purification

M9TG broth in 2‑liter baffled Erlenmeyer flasks was inoculated with *B. thailandensis* strains E555 or BT2683 and incubated overnight at 37 °C with shaking (200 rpm). Cell pellets were collected by centrifugation, resuspended in 50% ethanol (EtOH), and vigorously stirred for 30 min at room temperature. Insoluble material was removed by centrifugation, and the clarified extract was filtered through a 0.45 µm Rapid‑Flow bottle filter unit (Millipore). Absolute EtOH was added to the filtrate to a final concentration of 90% (v/v), and the mixture was left to stand at room temperature for 60 min to precipitate the CPS antigen. Insoluble material was collected by centrifugation and resuspended in dH_2_O. The solubilized material was dialyzed against dH₂O using a 3,500 MWCO G3 Slide‑A‑Lyzer cassette (Pierce) and lyophilized. Purified CPS was then obtained by mild acid hydrolysis and size‑exclusion chromatography, as previously described in refs. ^31,32^.

### Nuclear Magnetic Resonance (NMR) spectroscopy

Column-purified E555 and BT2683 CPS samples were dissolved in D_2_O (99.9% D, Aldrich) and lyophilized. Each sample was then re-dissolved in D_2_O (99.96% D, Cambridge Isotope Laboratories) and transferred to a 5-mm NMR tube. NMR data were acquired at 50 °C on a Varian VNMRS spectrometer (^1^H, 599.66 MHz) equipped with a cold probe. Quantitative ^1^H NMR spectra were acquired with a spectral width of 9615 Hz, 16384 complex data points, 8 transients and a total recovery delay of 62 s between the transients. ^1^H signal positions of the 6-deoxyheptan and mannan CPS carbohydrates matched the chemical shifts determined previously in ref. ^38^. Additionally, two-dimensional ^1^H,^1^H-COSY, ^1^H,^1^H-TOCSY, ^1^H,^1^H-NOESY, ^1^H,^13^C-HSQC, ^1^H,^13^C-HSQC-TOCSY and ^1^H,^13^C-HMBC were acquired to identify a low-abundance acetylated Rha component. ^1^H and ^13^C chemical shifts were referenced to the respective signals of acetone (2.22 and 33.0 ppm). The data were processed and analyzed in Mestrenova (version 14.1.1).

### Glycoconjugate synthesis

Recombinant, pre-clinical grade CRM197 was obtained from Fina Biosolutions. The CPS-CRM197 glycoconjugate used in this study was synthesized essentially as previously described in ref. ^21^. For control purposes, CRM197 alone or oxidized-CPS alone were incubated in the presence of NaBH₃CN at 37 °C with stirring for 7 days. A BCA assay (Pierce) was used to quantitate the amount of protein in the resulting material (with the remainder of the mass assumed to be polysaccharide). The amount of endotoxin in the glycoconjugate preparation was quantitated using a LAL Chromogenic Endotoxin Quantitation Kit (Pierce).

### Protein expression and purification

For expression of Hcp1-EK, the *hcp1* ORF (BMAA0742) was PCR amplified from *B*. *mallei* ATCC 23344 genomic DNA using the Bmhcp1-FXh and Bmhcp1-RH (Table 2) primer pair. The resulting DNA fragment was digested with XhoI and HindIII and cloned into pBAD/HisA, which was also digested with XhoI and HindIII, producing the plasmid pBADHisA-Bmhcp1-EK. Notably, *B*. *pseudomallei* and *B*. *mallei* Hcp1 proteins are 99.4% identical. Recombinant DNA techniques were conducted as previously described in ref. ^70^. Oligonucleotide primers were obtained from Integrated DNA Technologies. DNA sequencing was performed by ACGT Inc.

For purification of Hcp1-EK, *E*. *coli* TOP10 (pBADHisA-Bmhcp1-EK) was grown to mid-log phase in LBL broth and protein expression was induced using 0.02% L-arabinose. Bacterial pellets were resuspended in B-PER (Pierce) plus Benzonase (Novagen) and Lysozyme (100 μg/ml) and incubated for 20 min at room temperature with gentle agitation. Insoluble material was removed by centrifugation, and the resulting supernatant was loaded onto a gravity-fed His-Pur Cobalt Resin (Thermo Scientific) column. The column was washed with Wash Buffer (40 mM Tris pH 8.0, 300 mM NaCl and 40 mM Imidazole), and the protein was eluted with Elution Buffer (40 mM Tris pH 8.0, 50 mM NaCl and 300 mM Imidazole) then dialyzed against 1X EK Cleavage Buffer (20 mM Tris pH 7.5, 50 mM NaCl, and 2 mM CaCl_2_). To remove the His-Tag, purified Hcp1-EK was then incubated with various amounts of enterokinase (EK-His, Bovine, GenScript) at 37 °C for 20 h. For routine cleavage of Hcp1-EK, 200 U of EK-His was used per milligram of protein. Following this, an equal volume of Cobalt Loading Buffer (20 mM Tris pH 7.5, 250 mM NaCl) was added to the sample before loading onto a gravity-fed His-Pur Cobalt Resin column. The flow-through containing the resulting His-tagless Hcp1 (Hcp1-TL) was concentrated and dialyzed against PBS (BupH, pH 7.2, Thermo Scientific) using a 3500 MWCO Slide-A-Lyzer cassette (Thermo Scientific) at 4 °C. Endotoxin removal was performed using High-Capacity Endotoxin Removal Resin (Pierce) according to the manufacturer’s instructions. The Hcp1-TL samples were then sterilized with a syringe filter (0.22 µm) and stored at 4 °C. Protein concentrations were determined using a BCA protein assay kit (Pierce). The amount of endotoxin in the Hcp1-TL preparation was quantitated using a LAL Chromogenic Endotoxin Quantitation Kit (Pierce).

### SDS-PAGE and Western immunoblotting

Glycoconjugate and purified protein samples were diluted in 2x SDS-PAGE sample buffer to a final concentration of 1x, heated to 100 °C for 5 min, and electrophoresed on 4–12% or 12% Bis-Tris Bolt gels (Invitrogen), respectively. Proteins were visualized using SimplyBlue SafeStain (Life Technologies). For immunoblot analysis, the purified CPS or glycoconjugates were electrophoresed on 4–12% Bis-Tris Bolt gels (Invitrogen) and then transferred to a 0.45 µM pore-size nitrocellulose membrane (Life Technologies). The membranes were blocked with StartingBlock T20 (TBS) Blocking Buffer (Pierce) for 30 min at room temperature and then incubated with a 1/2000 dilution of a *B. pseudomallei* CPS-specific monoclonal antibody (mAb) 3C5 or *B. pseudomallei* OPS-specific mAb Pp-PS-W for 1 h at room temperature^44,71^. To facilitate the detection, the membranes were incubated for 1 h at room temperature with a 1/5000 dilution of anti-mouse IgG1-HRP conjugate (Southern Biotech). The blots were visualized with ECL Western Blotting substrate (Pierce) and imaged on a ChemiDoc imaging system (Bio-Rad).

### SEC analysis of CPS-CRM197 Glycoconjugates

FPLC-SEC was performed using an ÄKTA go system (Cytiva) to characterize the elution profiles of CRM197 and CPS-CRM197. CRM197, either as a glycoconjugate or as unconjugated CRM197 (control), were prepared in PBS and loaded onto a Superdex 200 Increase 10/300 GL high-resolution gel filtration column (Cytiva). Separation was carried out using PBS as the mobile phase at a flow rate of 0.75 mL/min under isocratic conditions. Protein elution was monitored by absorbance at 280 nm.

### Ethics statement

Immunizations and blood collections for the NHP were performed at Charles River Laboratories in Reno, Nevada, which is accredited by the Association for Assessment and Accreditation of Laboratory Animal Care. The protocol and procedures performed on the animals in this study were approved by Charles River-Nevada Institutional Animal Care and Use Committee (protocol no. 20263524 and 20337447) as well as by the U.S. Army Medical Research and Development Command Animal Care and Use Review Office (protocol no. CB-2019-04.02 and CB-2019-04.e001). The care and use of animals were conducted in accordance with established guidelines of the USA National Research Council and Animal Welfare Act regulations. Animals were acclimated to laboratory housing for at least 14 days prior to the initiation of dosing. They were monitored for physical health, body weight, food consumption, and clinical pathology throughout the study. Following the last blood collection, all animals were returned to the Testing Facility animal colony.

This study was designed as an exploratory, preliminary assessment of vaccine safety and immunogenicity in a very limited cohort of non-human primates (two males and two females per vaccine dose). Given the intentionally small group sizes, which were selected in accordance with the principles of reduction and responsible use of animals in early-stage research, the study is not powered to support formal statistical analyses or to comprehensively address all elements of the ARRIVE Essential 10 guidelines. Instead, the primary objective was to generate initial qualitative and trend-level data to inform the design of subsequent, adequately powered studies. As such, certain ARRIVE reporting items, particularly those relating to sample size justification, randomization, and statistical methods, cannot be meaningfully or rigorously addressed in this exploratory context.

### Cynomolgus macaque immunizations and blood collection

Two groups of two male and two female Asian-origin cynomolgus macaques, aged 2 to 4 years and weighing 3.5 to 6 kg at the time of initial dosing, were immunized s.c. in the scapular region on days 0, 21, and 35. The first group was immunized with the low-dose formulation: CPS-CRM197 (5 µg of CPS/dose as a conjugate) plus Hcp1-TL (10 µg/dose) formulated in PBS with 2% Alhydrogel (250 µg/dose which contained 88.4 µg of Al; Brenntag) and ODN 2006 (10 µg/dose) and the second group was immunized with the high-dose formulation: CPS-CRM197 (10 µg of CPS/dose as a conjugate) plus Hcp1-TL (20 µg/dose) formulated in PBS with 2% Alhydrogel (500 µg/dose which contained 176.8 µg of Al; Brenntag) and ODN 2006 (20 µg/dose; Invivogen). For plasma and PBMC isolation, blood was collected in K_3_EDTA tubes two weeks before the first dose and on days 7, 28, 42, 56, 70, and 84 post-immunization. For whole blood stimulation assays, blood was collected in sodium heparin tubes on days 28 and 42. No sedation was used for the immunizations, whereas sedation (10–15 mg/kg intramuscular ketamine) was used for the blood collections.

### Clinical chemistry, hematology and coagulation measurements

The safety of the vaccine formulations was monitored through complete blood analysis and daily health examinations. Samples for clinical chemistry analysis were collected into tubes without anticoagulant and measured using an Olympus AU640e^TM^ chemistry analyzer. Samples for hematology and coagulation analysis were collected in tubes containing K_3_EDTA and sodium citrate, respectively. Hematology and coagulation parameters were measured on a Siemens ADVIA® 120/2120 hematology analyzer and a STago STA Compact® coagulation analyzer, respectively. The blood samples were collected 2 weeks before immunization and at days 42 and 70 post-immunization. The clinical analysis included aspartate aminotransferase (AST), alanine aminotransferase (ALT), alkaline phosphatase (ALP), gamma-glutamyl transferase (GGT), lactate dehydrogenase (LDH), total bilirubin (TBIL), blood urea nitrogen (UREAN), creatinine (CREAT), glucose (GLUC), cholesterol (CHOL), triglyceride (TRIG), total protein (TPROT), albumin (ALB), globulin (GLOB), albumin/globulin ratio (A/G), calcium (CA), phosphorus (PHOS), sodium (Na), potassium (K) and chloride (Cl). Hematology and blood coagulation analysis included white blood cell count (WBC), neutrophil count (NEUT), lymphocyte count (LYMPH), monocyte count (MONO), eosinophil count (EOS), basophil count (BASO), large unstained cell (LUC), red blood cell count (RBC), hemoglobin (HGB), hematocrit (HCT), mean corpuscular volume (MCV), mean corpuscular hemoglobin (MCH), mean corpuscular hemoglobin concentration (MCHC), red cell distribution width (RDW), platelet count (PLT), reticulocyte count (RETIC), prothrombin time (PT), activated partial thromboplastin time (APTT) and fibrinogen (FIB).

### PBMC isolation

PBMCs were isolated from 15 ml of K_3_EDTA-treated blood by density-gradient centrifugation using 50 ml Sepmate™ tubes (Stemcell Technologies). Briefly, 15 ml of Lympholyte-Mammal Cell Separation Media (Cedarlane) was loaded into 50 ml Sepmate™ tubes. Whole blood diluted 1:1 with PBS (Gibco pH 7.2) was then added to the Sepmate™ tubes. Samples were centrifuged for 20 min at 1200 × g at 22 °C. The PBMC layer was then poured into a 50 ml conical tube and centrifuged at 300 × g for 8 min. The PBMCs were washed twice with 30 ml of Roswell Park Memorial Institute (RPMI) 1640 medium via centrifugation at 300 × g for 8 min The PBMCs were then slowly resuspended in RPMI 1640 medium containing 20% heat-inactivated fetal bovine serum (FBS, Gibco) and 10% dimethyl sulfoxide (Sigma), brought to −80 °C in a CoolCell (Corning) freezing container and subsequently transferred to a liquid nitrogen tank for storage until required for use.

### Analysis of antibody titers

Plasma samples were processed using K_3_EDTA tubes according to the manufacturer’s instructions and stored at −80 °C until required for use. Antibody responses against the vaccine antigens were assessed by ELISA. Briefly, 96-well Maxisorp plates (Nunc) were coated overnight at 4 °C with purified CPS or Hcp1-TL (0.1 µg/well) solubilized in carbonate buffer (pH 9.6). The plates were blocked at room temperature for 30 min with blocking buffer (3% skim milk (w/v) in Tris-buffered saline (Teknova) plus 0.05% Tween 20 (TBS-T)), washed three times with TBS-T, and then incubated with NHP plasma samples serially diluted in TBS-T plus 10% blocking buffer for 1 h at 37 °C. The plates were washed three times with TBS-T before incubation with 1/5,000 dilutions of anti-monkey IgG HRP-conjugated antibodies (Southern Biotech) for 1 h at 37 °C. The plates were washed four times with TBS-T and then developed with TMB (3,3’,5,5’-tetramethylbenzidine) substrate (KPL, Seracare). After 20 min of incubation at room temperature, stop solution (1 M HCl) was added to the wells and the plates were read at 450 nm using a FLUOstar Omega microplate reader (BMG Labtech). The reciprocals of the highest dilutions with optical densities (ODs) 3x background levels were used to determine the endpoint titers for the individual NHPs.

### THP-1 cell culture

The THP-1 cell line was obtained from the American Type Culture Collection (ATCC, TIB-202) and maintained in RPMI 1640 supplemented with 10% (vol/vol) heat-inactivated FBS (RPMI-10) in a humidified incubator at 37 °C with 5% CO_2_. THP-1 monocytes (1 × 10^6^ cells) were differentiated into macrophages in 24-well tissue culture-treated plates (Corning) using RPMI-10 and 5 ng/ml PMA for 2 days. Differentiated THP-1 cells were allowed to rest in fresh RPMI-10 for an additional 3 days before conducting OPC assays.

### Flow cytometry analysis of THP-1 differentiation

THP-1 cells were differentiated in 24-well tissue culture-treated plates as described above. The cell-surface markers of differentiated THP-1 cells were analyzed by flow cytometry before initiating the OPC assays. The differentiated THP-1 cells were pelleted and washed in PBS. Cells were then stained with viability 405/520 fixable dye, FITC-anti-human CD11b (Miltenyi Biotec clone REA713), PE-anti-human CD35 (Miltenyi Biotec clone REA1133) and APC-anti-human CD14 (Miltenyi Biotec clone REA599) and analyzed using a MACSQuant Analyzer 10 (Miltenyi Biotec). Data analysis was performed with FlowJo version 10 (FlowJo LLC, Ashland, OR, USA).

### OPC assays

OPC assays were performed using differentiated THP-1 cells as described above. Since *B. thailandensis* E555 is an avirulent strain and expresses the same CPS as *B. pseudomallei*, this strain was used as a surrogate in OPC assays, which allowed for experiments to be conducted using biosafety level 2 (BSL-2) containment and practices^72^. Briefly, *B. thailandensis* E555 cultures grown to early log phase were pelleted, resuspended at a density of 1 × 10^6^ CFU/ml in RPMI 1640 or RPMI 1640 containing 5% pre-immune plasma, day 28 plasma or day 84 plasma (heat-inactivated for 30 min at 56 °C) and then incubated at 37 °C for 1 h. The opsonized bacterial suspensions were added to differentiated THP-1 cells at an MOI of 0.1, incubated at 37 °C with 5% CO_2_ for 1 h, and then washed with RPMI 1640 medium to remove extracellular bacteria. Infected THP-1 cells were incubated with fresh RPMI-10 containing 500 μg/ml kanamycin for 2 h to suppress the growth of residual extracellular bacteria. The infected THP-1 cells were then washed twice with PBS, lysed with 0.2% (vol/vol) Triton X-100 (Sigma), and serial dilutions of the lysates were plated onto LBL agar plates and incubated at 37 °C for 48 h. Plate counts were used to enumerate bacterial loads. Three independent experiments were performed in duplicate.

### IFN-γ ELISpot assays

Cellular immune responses to protein antigens were assessed ex vivo using IFN-γ ELISpot assays with cryopreserved PBMCs. The 96-well ELISpot assays were performed using Human IFN-γ ImmunoSpot® kits (Cellular Technology Ltd.). Briefly, the plates were coated overnight with 80 µl of anti-human IFN-γ-antibody at 4 °C. The next day, plates were washed once with PBS, and antigens, including Hcp1-TL and CPS-CRM197, were added at a final concentration of 25 µg/ml. PBMCs were added in duplicate to wells of 96-well plates at 3 × 10^5^ PBMCs/well and incubated at 37 °C, 5% CO_2_ for 24 h. Following plate development, spot-forming units (SFU) in each well were counted using an ImmunoSpot S6 Micro Analyzer and ImmunoSpot v5.1 Professional DC Smart Count software (Cellular Technology Ltd.). Results were expressed as IFN-γ spot-forming cells (SFC) per million PBMCs. Background levels of cellular responses in unstimulated control wells were subtracted from those measured in antigen-stimulated wells. Phytohaemagglutinin (PHA, Sigma) was used as a positive control at a final concentration of 2.5 µg/ml to ensure T cell responsiveness.

### Whole blood stimulation assays

Whole blood was collected on days 28 and 42 in sodium heparin tubes and diluted 1:1 with RPMI-10 containing Pen-Strep. The diluted blood was then added to a 96-well plate (Corning) containing Hcp1-TL and CPS-CRM197 at a final concentration of 12.5 µg/ml and incubated at 37 °C in 5% CO_2_ for 48 h. PHA at a final concentration of 2.5 µg/ml and cell culture medium alone were used as positive and negative controls, respectively. The 96-well plate was centrifuged at 2000 × g for 15 min, and the supernatants were collected for cytokine analysis.

### IFN-γ ELISAs

Following whole blood stimulation, plasma IFN-γ levels for the day 28 and day 42 samples were measured in duplicate using a Monkey IFN-γ ELISA^pro^ kit (Mabtech) according to the manufacturer’s instructions. Briefly, the pre-coated strip plates were washed and incubated with 100 µl of Mabtech recombinant human IFN-γ or plasma sample diluted in ELISA diluent. After 2 h of incubation at room temperature, the plates were washed and incubated with 100 µl of the biotinylated detection antibody at a 1:1000 dilution for 1 h at room temperature. After washing, 100 µl of streptavidin-horseradish peroxidase diluted at 1:1000 was added to each well and incubated for 1 h at room temperature. The plates were developed using 100 µl of TMB substrate for 15 min in the dark at room temperature. The assays were stopped by adding 100 µl of H_2_SO_4_ 0.18 M and then read at 450 nm using a FLUOstar Omega microplate reader (BMG Labtech). The levels of IFN-γ were determined using a standard curve.

### Statistical analysis

All graphs were produced using GraphPad Prism version 9.4.1 (GraphPad Software, Inc.). OPC assays were analyzed using an unpaired t-test. P-values of ≤ 0.05 were considered significantly different.

## Supplementary information

Supplementary Information

## Data Availability

The data supporting the findings of this study are available within the article and its Supplementary Information files. The raw data files used to generate figures will be made available upon request from the corresponding author.
