# Supplementary material for: Safety and immunogenicity testing of a melioidosis subunit vaccine candidate in cynomolgus macaques
Source: NPJ Vaccines. 2026 Jul 14;11:207. doi: 10.1038/s41541-026-01526-5 (PMC13736900; doi:10.1038/s41541-026-01526-5)
Supplement: Supplementary file 1 [file 41541_2026_1526_MOESM1_ESM.pdf]

Supplementary information

Safety and Immunogenicity Testing of a Melioidosis Subunit Vaccine Candidate in Cynomolgus macaques

Sineenart Sengyee[1], Caitlyn E. Orne[1], Sarah B. Weiby[1], Lindsey K. Schmidt[1], Federico Urbano-Munoz[1], Jiri Vlach[2], Christian Heiss[2], Parastoo Azadi[2], Narisara Chantratita[3,4], Mary N. Burtnick[1, 3] and Paul J. Brett[1,3,*]

[1]Department of Microbiology and Immunology, University of Nevada, Reno School of Medicine, Reno, NV, USA

[2]Complex Carbohydrate Research Center, University of Georgia, Athens, GA, USA

[3]Department of Microbiology and Immunology, Faculty of Tropical Medicine, Mahidol University, Bangkok, Thailand

[4]Mahidol-Oxford Tropical Medicine Research Unit, Faculty of Tropical Medicine, Mahidol University, Bangkok, Thailand.

Supplementary Table 1. Assignment of ^{1}H and ^{13}C NMR signals of *B. thailandensis* BT2683 CPS glycans.

Letter	Residue		Atom chemical shift (ppm)							
			1	2	3	4	5	6	7	OAc
A	→3-(2-OAc)-6d-β-*man*Hep-1→	^{1}H	4.90	5.30	3.96	3.49	3.49	1.75,2.16	3.78	2.13
		^{13}C	98.8	72.1	81.5	71.7	75.5	36.3	60.9	22.8
B	→3-α-Man-1→	^{1}H	5.12	4.22	4.00	3.77	3.83	3.76,3.90		
		^{13}C	104.8	72.4	80.9	68.9	76.2	63.8		
C	→3-(2-OAc)-β-Rha-1→	^{1}H	4.90	5.30	3.95	3.41	3.51	1.35		2.13
		^{13}C	98.8	72.1	81.4	73.1	74.8	19.5		22.8

Supplementary Table 2. Average yield of nine independent CPS-CRM197 conjugation reactions.

Lot	Mass CPS (mg)	Mass CRM197 (mg)	Total starting (mg)	Total yield (mg)	% Recovery
**CC01056**	65.7	40.0	105.7	80.3	76.0
**CC29016**	64.4	40.0	104.4	86.9	83.2
**CC16016**	64.1	40.0	104.1	81.5	78.3
**CC11095**	63.7	40.0	103.7	77.9	75.1
**CC05065**	64.0	40.0	104.0	78.3	75.3
**CC29055**	64.2	40.0	104.2	90.2	86.6
**CC12035**	64.8	40.0	104.8	82.5	78.7
**CC19025**	64.1	40.0	104.1	85.0	81.7
**CC18025**	63.7	40.0	103.7	83.7	80.7
**Average**					**79.5**

Supplementary Table 3. Blood chemistry measurements in cynomolgus macaques following immunization with low-dose formulation (CPS-CRM197: 5 µg of CPS/dose as a conjugate; Hcp1-TL: 10 µg/dose; CpG: 10 µg/dose; 2% Alhydrogel 250 µg/dose). Blood was collected from animals on days -14, 42 and 70. CM, cynomolgus macaque male; CF, cynomolgus macaque female.

	Parameter (Reference interval)																			
Time point	AST (22-125 U/L)	ALT (16-139 U/L)	ALP (85-732 U/L)	GGT (30-194 U/L)	LDH (31-900 U/L)	TBIL (0.1-0.5 mg/dL)	UREAN (15-32 mg/dL)	CREAT (0.1-1.4 mg/dL)	GLUC (45-126 mg/dL)	CHOL (82-197 mg/dL)	TRIG (26-97 mg/dL)	TPROT (6.0-8.2 g/dL)	ALB (3.6-5.0 g/dL)	GLOB (2.2-3.9 g/dL)	A/G (1.0-1.9 ratio)	CA (9.2-11.4 mg/dL)	PHOS (3.1-7.3 mg/dL)	Na (143-155 mEq/L)	K (3.8-6.6 mEq/L)	Cl (101-114 mEq/L)
Day -14																				
CM 01	125	181	410	77	625	0.2	17	0.8	60	102	36	6.9	4.5	2.4	1.9	9.9	4.7	148	4.1	109
CM 02	87	45	637	63	617	0.1	26	0.7	69	109	69	6.6	3.9	2.7	1.4	10.1	3	148	4.1	111
CF 01	44	36	116	60	349	0.2	17	0.8	63	176	76	7.1	4.1	3	1.4	9.7	2.9	143	3.5	108
CF 02	48	72	228	46	367	0.5	17	0.7	70	110	29	7	4.4	2.6	1.7	9.8	4.6	145	3.9	109
Day 42																				
CM 01	48	137	504	73	414	0.1	14	0.7	76	120	43	7.2	4.5	2.7	1.7	10.4	6.3	145	4.4	107
CM 02	36	35	677	78	413	0.1	16	0.8	85	135	48	6.9	4.3	2.6	1.7	10.1	6.1	145	3.8	107
CF 01	20	27	104	57	237	0.1	17	0.9	46	156	55	6.8	3.9	2.9	1.3	9.7	2.9	145	3.9	109
CF 02	40	104	207	47	417	0.1	19	0.8	65	129	48	7.2	4.4	2.8	1.6	9.8	5	148	3.7	109
Day 70																				
CM 01	45	126	494	78	408	0.1	14	0.7	76	120	48	7.2	4.5	2.7	1.7	10.6	6.1	146	4.2	108
CM 02	31	27	697	74	350	0.1	18	0.8	93	132	55	6.8	4.2	2.6	1.6	10.1	5.8	145	3.9	107
CF 01	17	20	105	51	241	0.1	18	0.9	59	155	92	6.9	4	2.9	1.4	9.8	2.5	144	3.7	108
CF 02	20	50	210	43	202	0.1	18	0.7	67	134	38	6.9	4.2	2.7	1.6	9.8	4.7	142	3.6	108

Supplementary Table 4. Hematology and blood coagulation measurements in cynomolgus macaques following immunization with low-dose formulation (CPS-CRM197: 5 µg of CPS/dose as a conjugate; Hcp1-TL: 10 µg/dose; CpG: 10 µg/dose; 2% Alhydrogel 250 µg/dose). Blood was collected from animals on days -14, 42 and 70. CM, cynomolgus macaque male; CF, cynomolgus macaque female.

Time point	Parameter (Reference interval)																		
	WBC (6.9-25.1 $10^3/\mu L$)	NEUT (1.99-16.78 $10^3/\mu L$)	LYMPH (2.36-12.99 $10^3/\mu L$)	MONO (0.16-0.76 $10^3/\mu L$)	EOS (0.01-0.44 $10^3/\mu L$)	BASO (0.01-0.15 $10^3/\mu L$)	LUC (0.02-0.27 $10^3/\mu L$)	RBC (4.97-6.69 $10^3/\mu L$)	HGB (11.7-15.5 g/dL)	HCT (38.3-51.4 %)	MCV (68.9-85.9 fL)	MCH (21.5-25.6 pg)	MCHC (28.4-32.1 g/dL)	RDW (11.4-14.6 %)	PLT (252-664 $10^3/\mu L$)	RETIC (29-114 $10^9/L$)	PT (10.1-12.9 sec)	APTT (19.8-29.7 sec)	FIB (159-399 mg/dL)
Day -14																			
CM 01	7.6	5.3	1.99	0.25	0.02	0.02	0.02	5.83	14.1	44	75	24.2	32.1	12.7	455	51.1	11.6	22.5	263
CM 02	10.8	5.44	5.02	0.21	0.04	0.03	0.07	5.83	13.1	41	71	22.5	31.9	12.5	417	62.5	11.9	19.2	299
CF 01	8.06	3.47	4.19	0.29	0.07	0.01	0.03	6.16	13.4	44	72	21.8	30.3	13.8	269	64.3	11.4	22.5	246
CF 02	7.67	4.81	2.4	0.36	0.06	0.01	0.02	5.31	13.3	39	74	25	33.6	11.9	382	45.4	12	22.3	213
Day 42																			
CM 01	6.8	2.7	3.48	0.48	0.12	0.02	0.02	5.8	14	44	76	24.1	31.9	13	525	77	11.3	22.2	250
CM 02	10.8	3.4	6.88	0.26	0.17	0.05	0.04	6.11	13.4	42	69	21.9	31.6	11.8	427	82.3	11.2	19.7	254
CF 01	9	4.06	4.39	0.37	0.11	0.02	0.04	6.05	13.3	43	71	22.1	31.3	12.6	281	81.7	11.3	22.3	217
CF 02	9.68	6.6	2.54	0.26	0.25	0.01	0.01	5.48	13.2	41	75	24.2	32.3	11.8	363	66.9	11.1	22.3	194
Day 70																			
CM 01	5.95	2.17	3.15	0.54	0.06	0.01	0.02	5.97	14.4	47	79	24.2	30.5	13	551	70.7	12.1	24.9	259
CM 02	12.28	3.78	7.89	0.42	0.11	0.03	0.03	5.57	13.2	41	73	23.8	32.7	12.2	392	86.7	11.6	20.5	247
CF 01	8.54	2.88	5.12	0.44	0.08	0.01	0.01	6.3	13.6	46	73	21.5	29.4	12.8	329	97.5	11.5	24.6	198
CF 02	5.15	2.55	2.22	0.27	0.1	0.01	0.01	5.57	13.6	44	78	24.5	31.3	12.2	374	57.9	11.5	25	184

Supplementary Table 5. Individual body weight measurement (kg) in cynomolgus macaques following immunization with low-dose formulation (CPS-CRM197: 5 µg of CPS/dose as a conjugate; Hcp1-TL: 10 µg/dose; CpG: 10 µg/dose; 2% Alhydrogel 250 µg/dose). CM, cynomolgus macaque male; CF, cynomolgus macaque female.

Male	Day(s) relative to start date														
	-8	-1	7	14	21	28	35	42	49	56	63	70	77	84	91
CM 01	5.5	5.5	5.5	5.8	5.9	5.9	5.9	6.1	6.2	6.1	6.3	6.1	6.3	6.2	6.3
CM 02	4.2	4.3	4.2	4.3	4.4	4.5	4.4	4.5	4.5	4.4	4.4	4.4	4.4	4.4	4.5
Mean	4.85	4.90	4.85	5.05	5.15	5.20	5.15	5.30	5.35	5.25	5.35	5.25	5.35	5.30	5.40
SD	0.92	0.85	0.92	1.06	1.06	0.99	1.06	1.13	1.20	1.20	1.34	1.20	1.34	1.27	1.27
Female	Day(s) relative to start date														
	-8	-1	7	14	21	28	35	42	49	56	63	70	77	84	91
CF 01	4.2	4.3	4.3	4.3	4.4	4.6	4.4	4.5	4.6	4.5	4.6	4.6	4.6	4.6	4.6
CF 02	3.6	3.7	3.8	3.8	3.9	3.9	3.8	3.9	3.9	4.0	4.1	4.1	4.1	4.1	4.0
Mean	3.90	4.00	4.05	4.05	4.15	4.25	4.10	4.20	4.25	4.25	4.35	4.35	4.35	4.35	4.30
SD	0.42	0.42	0.35	0.35	0.35	0.49	0.42	0.42	0.49	0.35	0.35	0.35	0.35	0.35	0.42

Supplementary Table 6. Blood chemistry measurements in cynomolgus macaques following immunization with high-dose formulation (CPS-CRM197: 10 µg of CPS/dose as a conjugate; Hcp1-TL: 20 µg/dose; CpG: 20 µg/dose; 2% Alhydrogel 500 µg/dose). Blood was collected from animals on days -14, 42 and 70. CM, cynomolgus macaque male; CF, cynomolgus macaque female.

	Parameter (Reference interval)																		
Time point	AST (22-125 U/L)	ALT (16-139 U/L)	ALP (85-732 U/L)	GGT (30-194 U/L)	LDH (31-900 U/L)	TBIL (0.1-0.5 mg/dL)	UREAN (15-32 mg/dL)	CREAT (0.1-1.4 mg/dL)	GLUC (45-126 mg/dL)	CHOL (82-197 mg/dL)	TRIG (26-97 mg/dL)	TPROT (6.0-8.2 g/dL)	ALB (3.6-5.0 g/dL)	GLOB (2.2-3.9 g/dL)	A/G (1.0-1.9 ratio)	CA (9.2-11.4 mg/dL)	PHOS (3.1-7.3 mg/dL)	Na (143-155 mEq/L)	K (3.8-6.6 mEq/L)	Cl (101-114 mEq/L)
Day -14																			
CM 03	32	26	487	95	393	0.03	17	0.8	81	134	32	7.3	4.7	2.6	1.8	10.3	4.5	147	5	108
CM 04	29	26	888	65	295	0.05	23	0.8	94	109	89	7.4	4.4	3	1.5	9.5	4.1	147	4.1	109
CF 03	33	43	169	45	642	0.07	13	0.8	88	156	186	7.2	4.4	2.8	1.6	9.6	4.1	148	3.7	108
CF 04	43	75	181	48	247	0.12	20	0.5	64	132	51	7.4	4.2	3.2	1.3	9.9	2.8	147	4.2	112
Day 42																			
CM 03	31	26	365	85	346	0.05	15	1	88	145	26	7.5	4.9	2.6	1.9	10.2	6	150	4.6	108
CM 04	39	29	667	54	513	0.05	18	1	94	100	24	7.5	4.4	3.1	1.4	9.4	5.1	149	4.4	109
CF 03	32	37	131	42	563	0.08	19	0.7	61	155	105	6.7	4.1	2.6	1.6	9.4	3.9	150	4.1	113
CF 04	28	32	172	48	257	0.24	18	0.6	68	147	39	7.6	4.5	3.1	1.5	10.3	44	150	4.1	112
Day 70																			
CM 03	29	34	305	80	316	0.13	17	0.9	72	163	33	7.2	4.7	2.5	1.9	10.1	4.5	149	4.4	108
CM 04	32	25	589	47	352	0.16	23	1.0	70	103	34	6.7	3.9	2.8	1.4	8.8	3.8	148	4.0	108
CF 03	33	36	121	43	681	0.15	22	0.7	60	159	126	6.6	4.1	2.5	1.6	9.7	3.1	147	3.9	109
CF 04	32	25	160	45	273	0.25	19	0.6	68	140	50	7.4	4.3	3.1	1.4	10.2	3.7	147	4.0	110

Supplementary Table 7. Hematology and blood coagulation measurements in cynomolgus macaques following immunization with high-dose formulation (CPS-CRM197: 10 µg of CPS/dose as a conjugate; Hcp1-TL: 20 µg/dose; CpG: 20 µg/dose; 2% Alhydrogel 500 µg/dose). Blood was collected from animals on days -14, 42 and 70. CM, cynomolgus macaque male; CF, cynomolgus macaque female.

Time point	Parameter (Reference interval)																		
	WBC (6.9-25.1 10^3/µL)	NEUT (1.99-16.78 10^3/µL)	LYMPH (2.36-12.99 10^3/µL)	MONO (0.16-0.76 10^3/µL)	EOS (0.01-0.44 10^3/µL)	BASO (0.01-0.15 10^3/µL)	LUC (0.02-0.27 10^3/µL)	RBC (4.97-6.69 10^3/µL)	HGB (11.7-15.5 g/dL)	HCT (38.3-51.4 %)	MCV (68.9-85.9 fL)	MCH (21.5-25.6 pg)	MCHC (28.4-32.1 g/dL)	RDW (11.4-14.6 %)	PLT (252-664 10^3/µL)	RETIC (29-114 10^9/L)	PT (10.1-12.9 sec)	APTT (19.8-29.7 sec)	FIB (159-399 mg/dL)
Day -14																			
CM 03	14.81	9.75	3.94	0.76	0.30	0.02	0.04	5.58	14.1	44.8	80.3	25.2	31.4	12.5	749	102.3	11.7	21.9	239
CM 04	8.47	4.83	3.28	0.25	0.08	0.01	0.02	5.34	12.8	39.6	74.1	24.0	32.4	12.4	317	47.8	12.3	23.9	233
CF 03	12.4	9.53	2.28	0.54	0.03	0.01	0.01	5.3	12.6	40.4	76.1	23.8	31.3	13.3	417	64.6	10.7	22.5	213
CF 04	9.41	4.72	4.24	0.23	0.16	0.02	0.04	5.97	12.9	38.9	65.1	21.6	33.2	15.4	386	55.5	12.0	25.2	176
Day 42																			
CM 03	9.34	5.41	3.30	0.46	0.11	0.01	0.05	5.59	14.5	46.4	83.0	25.2	31.2	112.1	628	76.7	11.7	24.2	244
CM 04	6.97	3.81	2.88	0.15	0.09	0.01	0.02	5.35	12.9	41.8	78.0	24.0	30.8	12.3	300	53.1	11.7	45.3	272
CF 03	13.2	9.98	2.69	0.44	0.02	0.05	0.02	5.0	12.2	39.9	79.8	24.4	30.6	13.4	419	58.1	10.9	41.7	199
CF 04	7.08	3.52	3.22	0.17	0.13	0.01	0.04	5.66	12.6	39.9	70.6	22.3	31.6	14.3	417	65.8	11.7	41.1	175
Day 70																			
CM 03	11.46	8.02	2.94	0.40	0.06	0.01	0.03	5.40	13.7	44.4	82.2	25.4	30.8	12.2	638	96.2	11.6	23.0	264
CM 04	5.88	3.83	1.91	0.10	0.03	0.01	0.01	5.25	12.7	40.7	77.4	24.2	31.3	12.0	302	40.7	12.3	25.5	239
CF 03	4.49	2.20	2.06	0.18	0.01	0.01	0.02	4.97	11.7	39.3	79.0	23.6	29.9	13.1	432	69.2	10.9	23.1	200
CF 04	5.61	2.80	2.59	0.11	0.08	0.01	0.02	5.5	12.4	39.0	70.9	22.5	31.8	14.0	416	80.3	11.4	26.3	203

Supplementary Table 8. Individual body weight measurement (kg) in cynomolgus macaques following immunization with high-dose formulation (CPS-CRM197: 10 µg of CPS/dose as a conjugate; Hcp1-TL: 20 µg/dose; CpG: 20 µg/dose; 2% Alhydrogel 500 µg/dose). CM, cynomolgus macaque male; CF, cynomolgus macaque female.

Male	Day(s) relative to start date														
	-8	-1	7	14	21	28	35	42	49	56	63	70	77	84	91
CM 03	5.2	5.3	5.4	5.4	5.5	5.4	5.4	5.4	5.4	5.3	5.3	5.3	5.2	5.1	5.2
CM 04	5.0	5.0	5.1	5.1	5.0	5.1	5.0	5.1	5.1	5.2	5.2	5.2	5.2	5.2	5.4
Mean	5.10	5.15	5.25	5.25	5.25	5.25	5.20	5.25	5.25	5.25	5.25	5.25	5.20	5.15	5.30
SD	0.14	0.21	0.21	0.21	0.35	0.21	0.28	0.21	0.21	0.07	0.07	0.07	0.00	0.07	0.14

Female	Day(s) relative to start date														
	-8	-1	7	14	21	28	35	42	49	56	63	70	77	84	91
CF 03	3.5	3.5	3.5	3.4	3.5	3.4	3.4	3.4	3.4	3.4	3.4	3.4	3.4	3.4	3.4
CF 04	3.6	3.6	3.7	3.6	3.7	3.8	3.8	3.8	3.8	3.8	3.7	3.8	3.8	3.8	3.8
Mean	3.55	3.55	3.60	3.50	3.60	3.60	3.60	3.60	3.60	3.60	3.55	3.60	3.60	3.60	3.60
SD	0.07	0.07	0.14	0.14	0.14	0.28	0.28	0.28	0.28	0.28	0.21	0.28	0.28	00.28	0.28

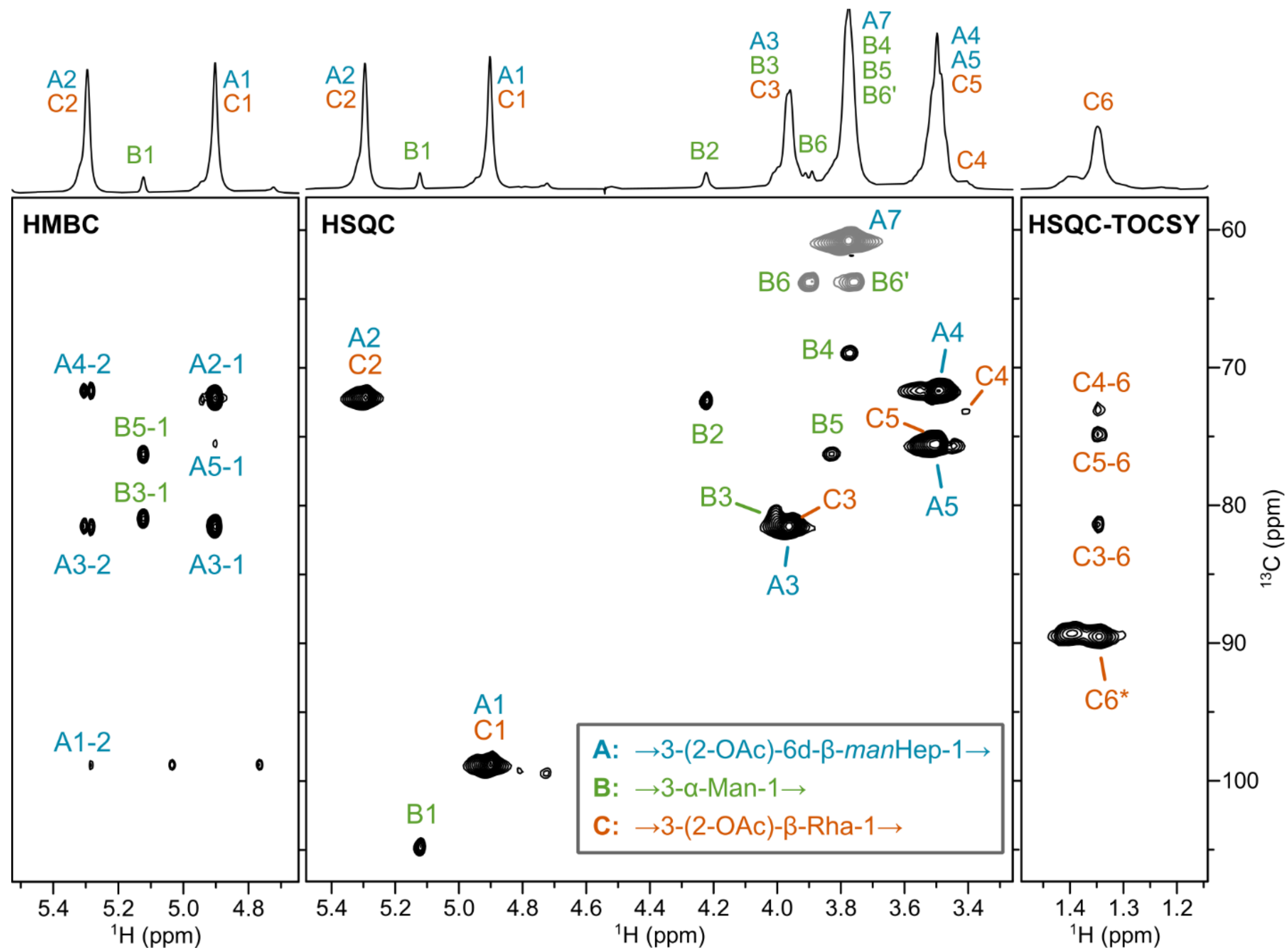

Supplementary Figure 1. Identification of low-abundance (2-OAc)-β-Rha residue illustrated on the analysis of ^{1}H,^{13}C-HMBC, ^{1}H,^{13}C-HSQC and ^{1}H,^{13}C-HSQC-TOCSY spectra. Signals are labeled using separate colors for the 6-deoxyheptan (blue), mannan (green) and Rha (orange). In the correlation spectra (HMBC, HSQC-TOCSY), each label consists of the locants of the interacting ^{13}C/^{1}H or ^{1}H/^{1}H atom pairs. The HSQC-TOCSY spectrum was acquired with a reduced spectral width and the C6* signal is aliased by 70 ppm. Most of the Rha signals overlapped with the signals of the dominant 6dHep but Rha-characteristic correlations were resolved in the HSQC-TOCSY spectrum. Due to signal overlaps, it was not possible to determine if the (2-OAc)-β-Rha residues formed a separate β-(1→3)-linked rhamnan polymer or if they were part of the 6-deoxyheptan polymer.

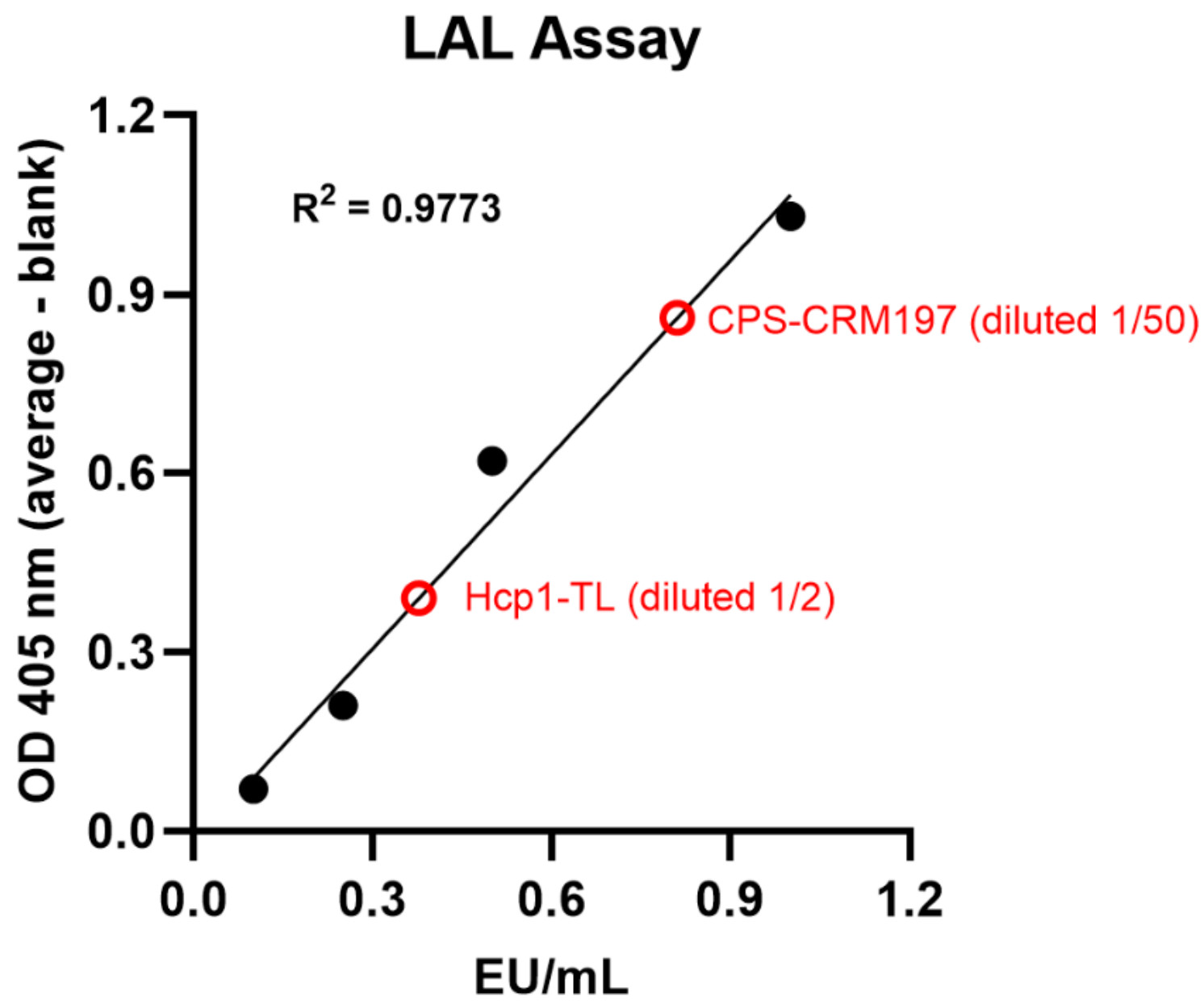

Supplementary Figure 2. Determination of endotoxin levels associated with the Hcp1-TL and CPS-CRM197 antigens used in this study. 1 mg/ml stocks of Hcp1-TL and CPS-CRM197 were diluted 1/2 and 1/50, respectively, with LAL water and assayed using a LAL Chromogenic Endotoxin Quantitation Kit (Pierce). Values were interpolated from the standard curve, indicating endotoxin levels of <1 EU/mg for Hcp1-TL and ~40 EU/mg for CPS-CRM197. EU = endotoxin units.

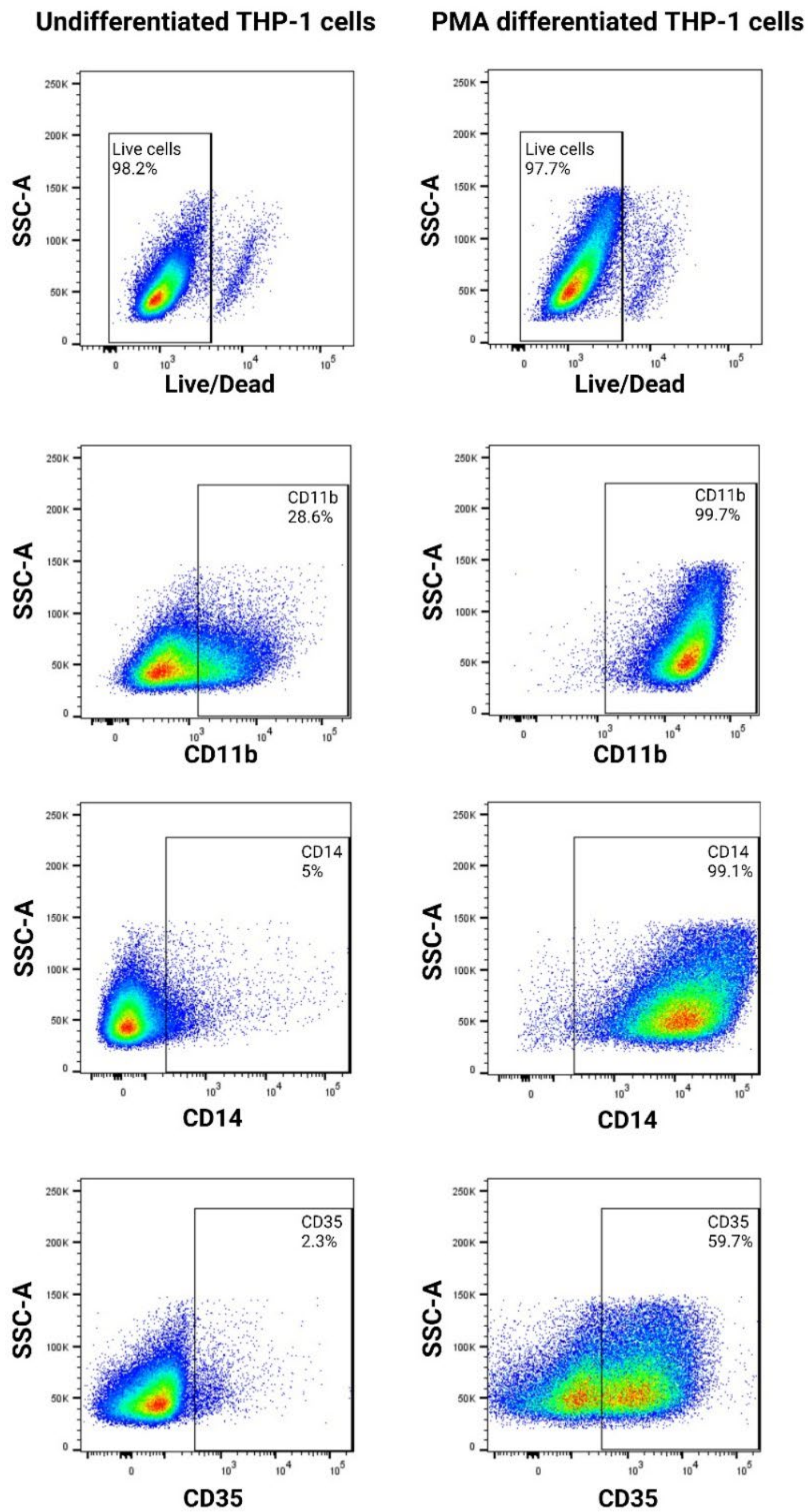

Supplementary Figure 3. Percentage of live, CD11b$^+$, CD14$^+$, CD35$^+$ in undifferentiated monocyte versus PMA-differentiated THP-1 macrophage cells. THP-1 cells increased the expression of CD11b, CD14 and CD35 after treatment with 5 ng/ml PMA for 48 hours.

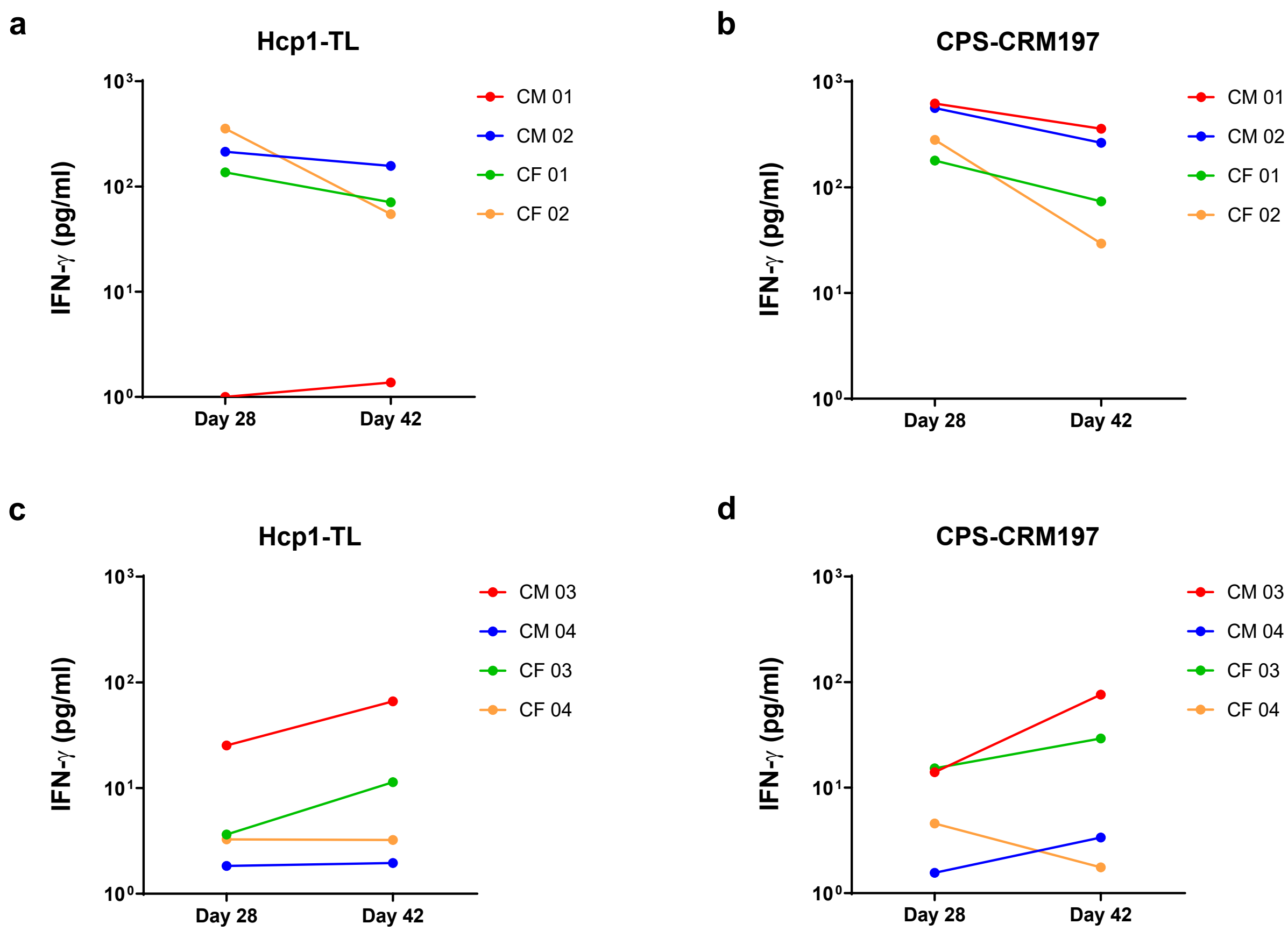

Supplementary Figure 4. Analysis of IFN-γ responses against Hcp1-TL and CPS-CRM197. Cynomolgus macaques (n = 4 per group) were immunized s.c. on days 0, 21, and 35 with CPS-CRM197 plus Hcp1-TL. Whole blood samples were collected one week after the second dose (day 28) and one week after the third dose (day 42) and stimulated with Hcp1-TL and CPS-CRM197 for 48 hours. IFN-γ responses were quantitated using Monkey IFN-γ ELISA^{pro} kits. (A) and (B) IFN-γ responses against Hcp1-TL and CPS-CRM197, respectively, at day 28 and day 42 post-immunization with low-dose formulation. (C) and (D) IFN-γ responses against Hcp1-TL and CPS-CRM197, respectively, at day 28 and day 42 post-immunization with high-dose formulation. Each dot represents the mean results of assays performed in duplicate for each animal. CM, cynomolgus macaque male; CF, cynomolgus macaque female.
